# Preparation and kinetic studies of a new antibacterial sodium alginate gelatin hydrogel composite

**DOI:** 10.1038/s41598-024-80453-8

**Published:** 2024-11-25

**Authors:** Reem A. ElTatawy, Amel M. Ismail, Mohammed Salah Ayoup, Magda M. F. Ismail, Howida Abouel Fetouh

**Affiliations:** 1 Department of Chemistry, Faculty of Science, Alexandria University, Alexandria, 21321 Egypt; 2 Department of Chemistry, College of Science, King Faisal University, Al-Ahsa, 31982 Saudi Arabia; 3 Department of Pharmaceutical Medicinal Chemistry, Faculty of Pharmacy (Girls), Al-Azhar University, Cairo, 11651 Egypt

**Keywords:** Drug delivery, Pseudo-second-order, 1, 2, 4-oxadiazole, Antibacterial, Spectroscopy, Biological techniques, Chemical biology, Chemistry, Materials science

## Abstract

This study involved synthesis of a novel antibacterial heterocyclic compound, sodium 2-(2-(3-phenyl-1, 2, 4-oxadiazol-5-yl) phenoxy) acetate abbreviated as Na-POPA. Further development of a biocompatible, pH-responsive hydrogel drug carrier prepared utilizing the natural polymers gelatin and sodium alginate. The compound loaded on the hydrogel represented new drug delivery system. Comprehensive characterization of Na-POPA was performed using Fourier-transform infrared spectroscopy (FT-IR), proton nuclear magnetic resonance (¹H NMR), carbon-13 nuclear magnetic resonance (¹³C NMR), and high-resolution mass spectrometry (HRMS). The compound was loaded onto the sodium alginate/gelatin hydrogel carrier under feasible experimental conditions. The successful incorporation of Na-POPA into the hydrogel matrix was confirmed via scanning electron microscopy (SEM), powder X-ray diffraction (pXRD) analysis and FT-IR spectroscopy. Cytotoxicity assays revealed that the all the loaded and unloaded compound induced cell toxicity at large concentration much lower than many reported results. The hydrogel reduced the inherent cytotoxicity of Na-POPA and enhanced its biocompatibility. The release kinetics of Na-POPA from the hydrogel were evaluated spectrophotometrically at different pH conditions simulating biological fluids. The release rate at pH 1.2 was greater than the release at pH 6.8, with a higher cumulative release observed at pH 6.8. The release kinetics obeyed the pseudo-second-order kinetic model, indicating a controlled release mechanism influenced by the hydrogel’s physicochemical properties. Electrochemical impedance spectroscopy and cyclic voltammetry further confirmed that the compound release was pH-dependent. The high swelling and solubility at pH 6.8 enhance the release. The larger amount released at 6.8 (target intestine) because of more solubility, leaching and swelling rather than shrinking.

**Supplementary Information:**

The online version contains supplementary material available at 10.1038/s41598-024-80453-8.

## Introduction

Since recognized antibiotics’ potential for disease treatment is dwindling, the public has been concerned about the rising bacterial resistance to these drugs^1,2^. As a result, efforts to synthesize and assess heterocyclic derivatives with antibacterial activity including 1, 2, 4-oxadiazoles have been increased^3,4^. The 1,2,4-oxadiazole heterocycle was initially created in 1884^5^, but the scientific community didn’t become interested in it until the 1990s^6^. The literature listed the 1,2,4-oxadiazole core’s had numerous medicinal uses, including antibacterial^7^, antitumor^8^, anti-Alzheimer^9^, anti-asthmatic^10^, anti-diabetic^11^, anti-inflammatory, and antioxidant^12,13^.

**Fig. 1:**
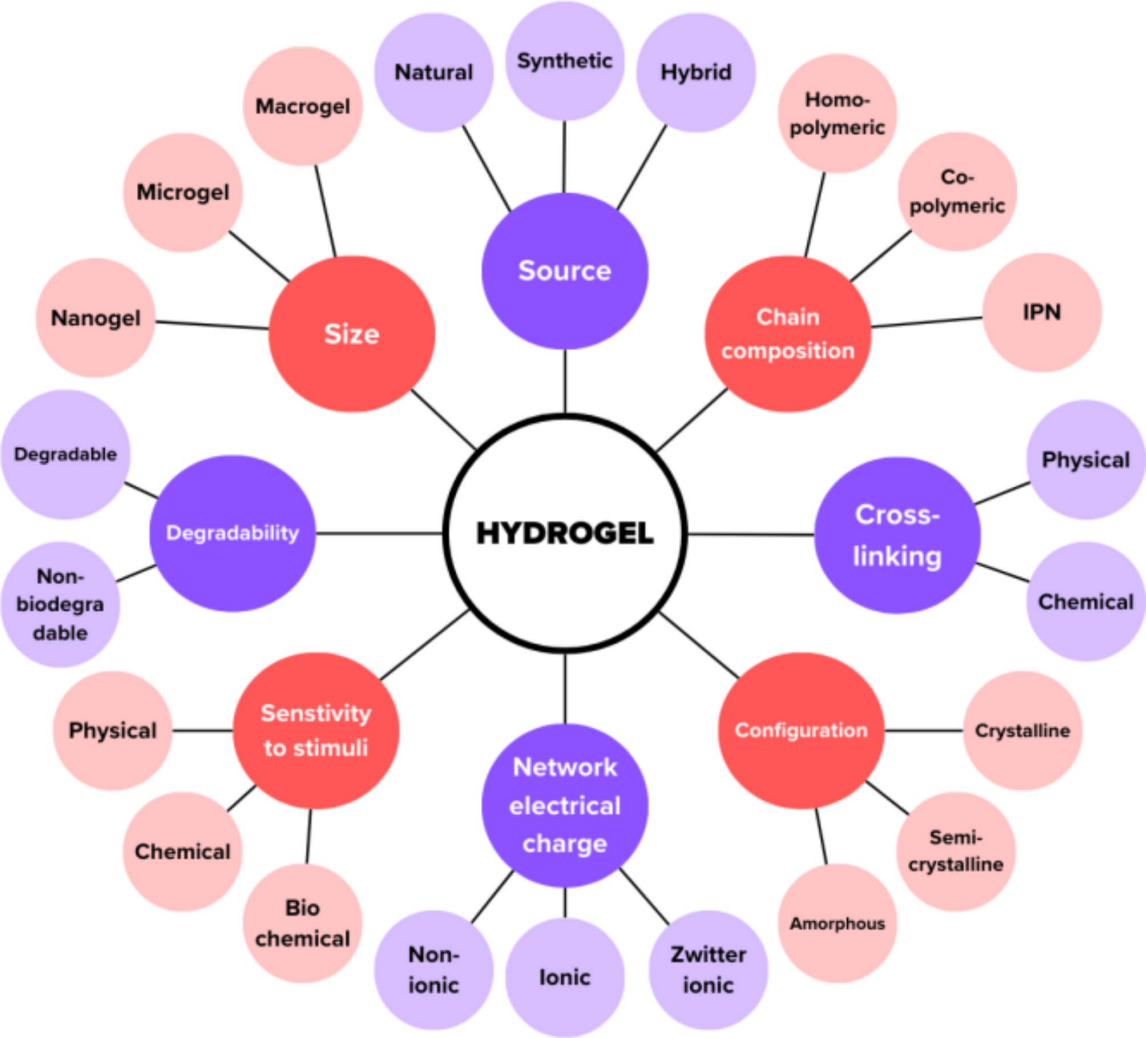
Schematic representation of hydrogel classifications.

One of the most intriguing areas of scientific research has been provided by polymeric drug delivery systems (DDS) like hydrogels. A pH-responsive hydrogel for the controlled release of antibacterial drugs is one of various strategies in the design and development of polymeric DDS. The pH-dependent swelling dynamics and drug release behavior from such hydrogels improve absorption and increase the bioavailability of several drugs inside the human body^14^. Hydrogels are three-dimensional hydrophilic polymeric networks and can be classified based on several criteria as summarized in Fig. 1^15–19^.

Generally, hydrogels are characterized by their unique ability to absorb large amounts of water causing swelling. This ability is attributed to the presence of hydrophilic groups along the polymeric backbone. In swollen state, hydrogels become rubbery soft and highly resemble biological tissues, making them excellent candidates for drug carrier^20^. However, the vast majority of traditional hydrogels usually have inhomogeneous morphology, poor mechanical strength, minimal swelling at equilibrium as well as poor response to pH and the temperature stimuli^21^.

Therefore, novel methods have been developed to overcome these inherent critical limitations and to prepare hydrogels with desirable characteristics and unique properties. Among the numerous approaches in the synthesis of hydrogels is using two distinct biodegradable and biocompatible polymers have the tendency for chemical modification^10,13,22^. Such hydrogels are suitable for the incorporation of drugs or bioactive factors and provide highly controlled release of the encapsulated drug. Moreover, these hydrogels are characterized by having remarkable physical properties that can be finely tuned, optimal swelling dynamics, non-toxicity, excellent biocompatibility and biodegradability that matches the rate of tissue regeneration^23^. Gelatin and alginate natural polymers are among the various polymers that have been combined in hydrogels fabrication.

Gelatin is one of the most versatile naturally occurring polymer. It is characterized by its biodegradability, biocompatibility and low immunogenicity. It is Generally Recognized as Safe (GRAS) by the United States Food and Drug Administration (FDA)^24^ and is therefore widely used in food, pharmaceutical, medical and cosmetic products^18^. It is obtained by the partial hydrolysis of the triple helix of collagen, the main protein constituent in skin, bones, connective tissues and hides of different animals. Much like collagen, gelatin is primarily made up of repeating triplet amino acid sequences of the glycine-proline-hydroxyproline^24^. Despite having several desirable features, gelatin has poor mechanical strength as well as poor stability. Moreover, at temperatures above 40℃, gelatin becomes water soluble and changes from gel state to sol state. Therefore, gelatin alone is rarely used on in hydrogel manufacturing. Gelatin is often cross-linked with other polymers to enhance its physicochemical properties^18^.

Among these polymers, alginate is a naturally occurring anionic linear polysaccharide that is often extracted from cell walls of algae biomass^25,26^ and certain bacterial species^18,25,26^. Alginate chains are composed of 1–4 linked α-L-guluronic acid (G) and β-D-mannuronic acid (M) units arranged in the form of tri blocks polymer. These blocks consist of successive G residues, successive M residues and alternating G and M residues^18,25–28^. Owing to its natural origin, alginate is considered to be biocompatible, biodegradable and mostly non-toxic, making it an ideal candidate to be used in the biomedical field, especially in DDS^18^. Several alginate-based hydrogels have been prepared and extensively studied, many of which are pH-responsive hydrogels^29–31^. However, most alginate-based hydrogels require the use of metal ions such as Ca^2+^ as cross linker for alginate chains. Despite the improved mechanical strength of hydrogels, the high dependence on the ionic environment limited their application in biomedicine^18,25,32^.

The addressed the problems that prompted this research is the wide spread of pathogenic bacteria and antibiotic resistant bacteria. This study aims preparation of new antibacterial heterocyclic compound and loading it on safe available biocompatible carrier.

**Fig. 2:**
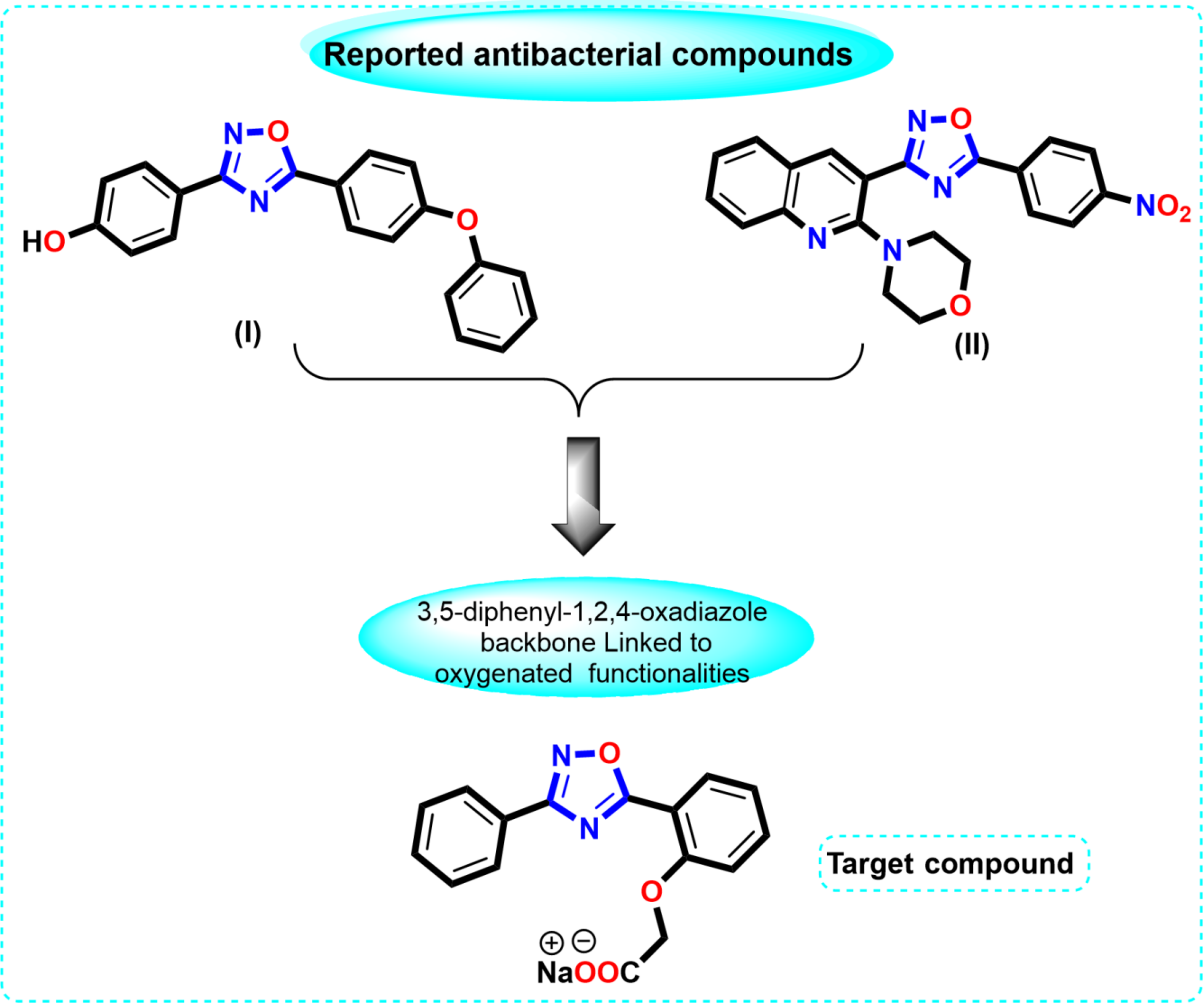
Feature similarities between leads I, II, and target compound.

The novelty of this current work is the synthesis of a new heterocyclic compound (Na-POPA) which demonstrated promising antibacterial activity. The Na-POPA was loaded on biocompatible hydrogel. Conducted comprehensive release kinetics to explore release amount and mechanism from the hydrogel matrix. Comparing the release kinetic by the conventional UV spectroscopy and electrochemical techniques could make this research relevance to the scientific community. The research protocol is to synthesize and explore the antibacterial activity of a new 1,2,4-oxadiazole derivative, namely sodium 2-(2-(3-phenyl-1,2,4-oxadiazol-5-yl)phenoxy)acetate (Na-POPA), following the feature similarities between the reported antibacterial leads **(I)**^33^, **(II)**^6,34^ and our target compound (Fig. 2).

The compound demonstrated adequate antibacterial activity against both Gram-positive and Gram-negative bacteria, making it a favorable candidate in combating antibiotics resistant bacterial. We also describe the loading of this promising antibacterial compound onto a sodium alginate/gelatin (SA/G) hydrogel carrier to achieve controlled and localized release in aqueous buffer solution simulating the biological fluids in the human body. The hydrogel carrier consisted only of two physically cross-linked biodegradable natural polymers (sodium alginate and gelatin) for rapid clearance from the human body after drug release.

## Experimental

### Materials and equipment

The materials and equipment are reported in the supplementary information (SI) section SI.1.

### Preparation of the biologically active compound (Na-POPA)

#### General method for synthesis of the amidoxime (2)

A mixture of hydroxylamine hydrochloride (0.015 mol) and sodium bicarbonate (0.015 mol) in absolute ethanol (50.0 mL) was refluxed for 15 min. with continuous stirring. Consequently, benzonitrile **1** was added, and the mixture was refluxed for an additional 8 h with continuous stirring. The resulting mixture was filtered and evaporated under reduced pressure, yielding the corresponding amidoxime **2**, which was used without further purification.

#### 2-(3-phenyl-1, 2, 4-oxadiazol-5-yl) phenol (3)

A mixture of the amidoxime **2** (0.0294 mol), methyl salicylate (0.0440 mol) and NaOH (0.1175 mol) in DMSO (10 mL) was stirred for 24 h, then the mixture was poured on cold water. The resulting precipitate was filtered to give **3**.

#### Ethyl 2-(2-(3-phenyl-1, 2, 4-oxadiazol-5-yl) phenoxy) acetate (4)

A mixture of **3** (0.0063 mol) and potassium carbonate (0.0289 mol) in dry acetone (50 mL) was refluxed with stirring for 15 min., then ethyl bromoacetate (1 mL) and potassium iodide were added to the mixture and further refluxed with stirring for 8 h. The resulting mixture was poured on cold water and the resulting precipitate was filtered to give **4**.

#### Sodium 2-(2-(3-phenyl-1, 2, 4-oxadiazol-5-yl) phenoxy) acetate (5) (Na-POPA)

A mixture of **4** (0.025 mol) and NaOH (0.025 mol) in THF/H_2_O (1:1) was refluxed for 1 h, then the reaction mixture was poured on cold water. The precipitate formed was filtered to give **5** as a white powder. IR (KBr): ʋ_max_/cm^-1^ 3067.54, 2939.21 (CH), 1599.79 (C = N), 1242.71 (Ar-O-CH_2_); ^**1**^**H NMR** (500 MHz, *DMSO-d*_*6*_) *δ*_*H*_: 4.34 (s, 2 H, C**H**_**2**_), 6.98 (d, J = 8.5 Hz, 1 H, Ar-**H**), 7.03 (t, J = 7.5 Hz, 1 H, Ar-**H**), 7.53 (t, J = 8.5 Hz, 2 H, Ar-**H**), 7.57 (d, J = 6.0 Hz, 2 H, Ar-**H**), 8.01 (d, J = 8.0 Hz, 1 H, Ar-**H**), 8.05–8.07 (m, 2 H, Ar-**H**); ^**13**^**C NMR** (125 MHz, *DMSO-d*_*6*_) *δ*_*C*_: 68.8 (O**C**H_2_), 112.5, 114.6, 120.3, 126.9, 127.6, 129.7, 131.4, 132.0, 134.6, 158.9 (Ar-**C**), 167.9 (O-**C** = O), 170.5, 176.1 (***Oxadiazol***-**C**); HRMS (m/z): [M]^+^calcd for [C_16_H_11_N_2_O_4_Na]^+^ gram mol^-1^: 319.06947 and found 319.06912.

### Preparation of SA/G hydrogel and SA/G/Na-POPA hydrogel

#### Preparation of 10% (w/v) gelatin solution

Gelatin (10 g) was added to distilled water (50 mL), then the volume of the solution was adjusted to 100 mL with double distilled water. The solution was heated in a water bath at 45℃ for 50 min^15^.

#### Preparation of SA/G hydrogel

Sodium alginate (0.9 g) was added to the gelatin solution (30 mL), and the mixture was heated in a water bath at 40℃ for 24 h with continuous stirring. The SA/G solution was then poured into a square silicon mold and allowed to stand at room temperature for 24 h for curing and forming the SA/G hydrogel^19^.

#### Preparation of SA/G/Na-POPA hydrogel

Similarly, sodium alginate (0.9 g) and Na-POPA (0.06 g) were added to the gelatin solution (30 mL), and the mixture was heated in a water bath at 40℃ for 24 h with continuous stirring. The solution was then poured into a square silicon mold and allowed to stand at room temperature for 24 h to form the SA/G/Na-POPA hydrogel^21^.

### Biological activity

The details of the biological activity tests (antibacterial and cytotoxicity assays) of Na-POPA, SA/G hydrogel, and SA/G/Na-POPA hydrogel are summarized in the supplementary information section SI.2 and SI.3^2,6,21^.

### In vitro release study

The release profiles of Na-POPA from the SA/G/Na-POPA hydrogel were studied spectrophotometrically using a UV-Visible spectrophotometer. 0.05 g of the SA/G/Na-POPA hydrogel were placed in 25 mL of the desired buffer (pH 1.2 or pH 6.8) simulating physiological fluids of stomach and colon. The time started on once the DDS immersed in the buffer solution. The reaction bottle contained the DDS-buffer solution mixture were shaken using waterbath shaker at 37 ± 0.5 ℃ and 100 rpm^21^. The amount of Na-POPA released from the hydrogel was studied *via* withdrawing aliquots of the supernatant at different scheduled time intervals and measuring the absorbance at λ_max_ 245 nm. The same volume of buffer at the same temperature was then added to the test mixture to maintain constant release volume (25 mL)^21^.

### Electrochemical monitoring of drug release

Electrochemical impedance spectroscopy (EIS) at open circuit potential and cyclic voltammetry (CV) at wide range potential window were used to confirm the release of Na-POPA from the hydrogel matrix. Both EIS and CV were carried out at 37℃ at 100 rpm. The common electrochemical cell contained: graphite working electrode (WE), Ag/AgCl_(s)_/KCl_(sat.)_ reference electrode (RE) and Pt. counter electrode (CE) in new test solutions have the same chemical compositions as that used in spectrophometric measurement. The cell was connected to the Gamry Potentiostat (USA) reference 600-Sequencer V6.20 software program. Graphite surface was activated by pure zeolite-water emulsion. After establishing the steady-state equilibrium potential of WE, the EIS test (at the open circuit potential of WE after steady state equilibrium for 15 min.) was conducted followed by CV. Software Gamry Echem Analyst Version 6.20 was used for data analysis^21,22^.

## Results and discussion

### Synthesis of sodium 2-(2-(3-phenyl-1, 2, 4-oxadiazol-5-yl) phenoxy) acetate

**Fig. 3:**
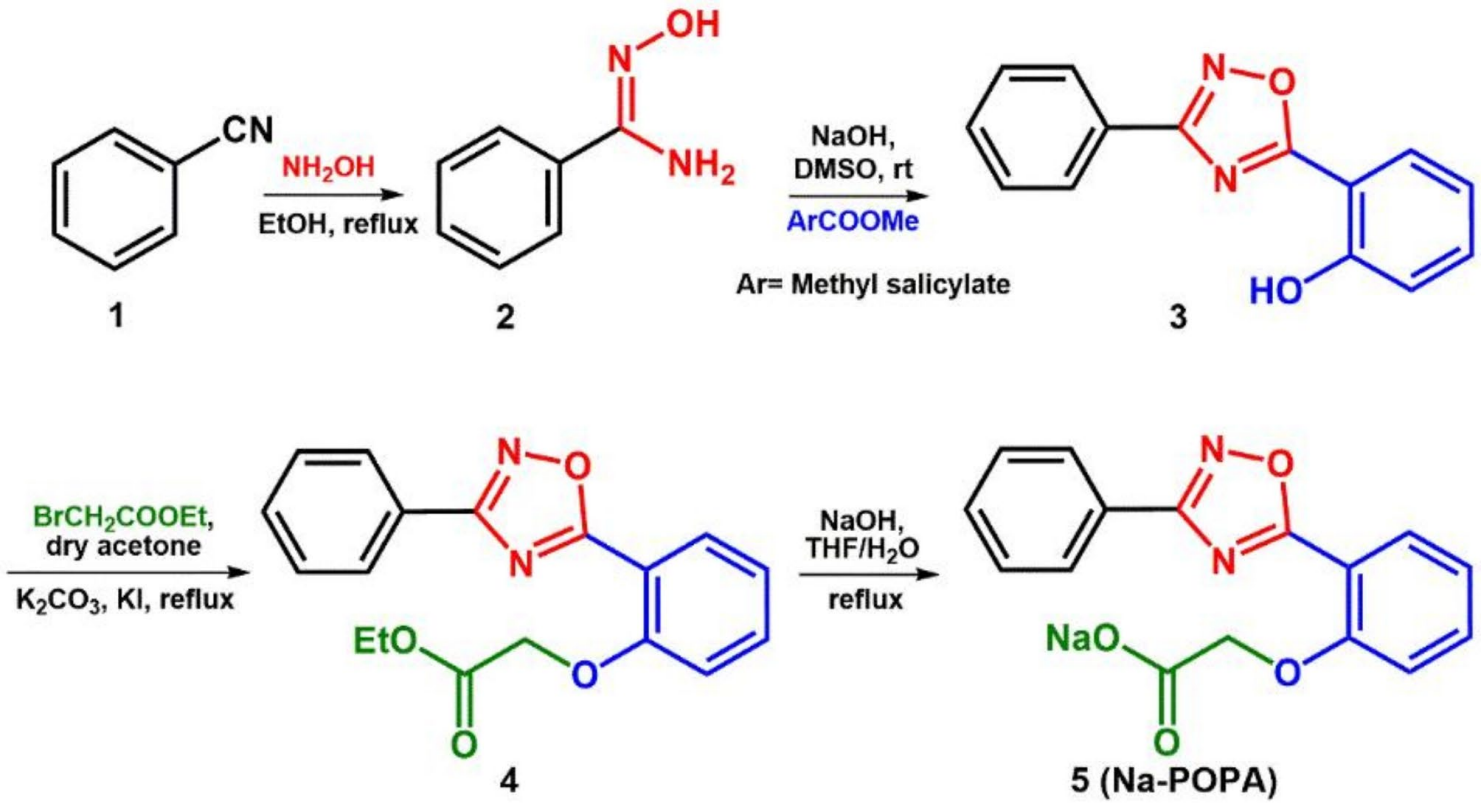
Synthesis of sodium 2-(2-(3-phenyl-1, 2, 4-oxadiazol-5-yl) phenoxy) acetate **(Na-POPA)**.

The 3,5-disubstituted-1,2,4-oxadiazole (**Na-POPA**) was prepared as described in Fig. 3*via* the reaction of benzonitrile **1** with hydroxylamine solution in ethanol, resulting in the respective amidoxime **2**. Afterwards, condensation of the amidoxime **2** with methyl salicylate using NaOH/DMSO gave the corresponding 3, 5-disubstituted-1, 2, 4-oxadiazole **3**. The 3, 5-disubstituted-1, 2, 4-oxadiazole **3** was then refluxed with ethyl bromoacetate in dry acetone in the presence of potassium iodide as a catalyst to give the corresponding ethyl ester **4**. Finally, the reaction of the ester **4** with sodium hydroxide in THF/H_2_O yielded the sodium salt of the 3, 5-disubstituted-1, 2, 4-oxadiazole **5**. The structure of the prepared compound **5** was confirmed by spectral analysis (section SI.4). The ^1^HNMR (Figs. SI.1, SI.2) showed characteristic signals for the CH_2_ groups at chemical shift 4.34 ppm and for the aromatic protons at chemical shift range 6.98–8.07 ppm, whereas the ^13^C NMR spectra (Figs. SI.3, SI.4) confirmed the presence of the carbonyl carbon at 167.9 ppm and the oxadiazole carbons at 170.5-176.1 ppm.

### Preparation of SA/G hydrogel

The sodium alginate/gelatin hydrogel was prepared according to a previously reported method with slight modifications^32^. Briefly, a mixture of 10% (w/v) gelatin solution and sodium alginate (1:0.03) was heated overnight at 40^o^C with continuous stirring at 100 rpm to form a uniform solution. The solution was then poured into a mold and cooled at room temperature to form the SA/G hydrogel. This SA/G hydrogel served as a blank hydrogel in the further tests. As opposed to the reference cited method, the synthesis of the SA/G hydrogel relied only on physical cross-linking and no additional cross-linking steps were performed. Physically cross-linked hydrogels are especially preferred in biomedical and drug delivery systems since they pose fewer risks of toxicity and are more biocompatible and biodegradable^35^.

### Preparation of SA/G/Na-POPA hydrogel

Conventionally, drugs and biologically active compounds are loaded onto hydrogels by soaking the blank hydrogel in the drug solution. However, a major drawback in this method is the uneven distribution of the drug in the hydrogel matrix, which leads to inaccurate drug release data. Therefore, an alternative batch synthesis method was employed during the preparation of the SA/G/Na-POPA hydrogel. For elucidation, Na-POPA was added to the SA/G mixture prior to the heating step. This ensures the formation of a homogenously loaded hydrogel with an even distribution of the target compound^15,21,36^.

### Fourier transform infrared spectroscopy (FTIR)

**Fig. 4:**
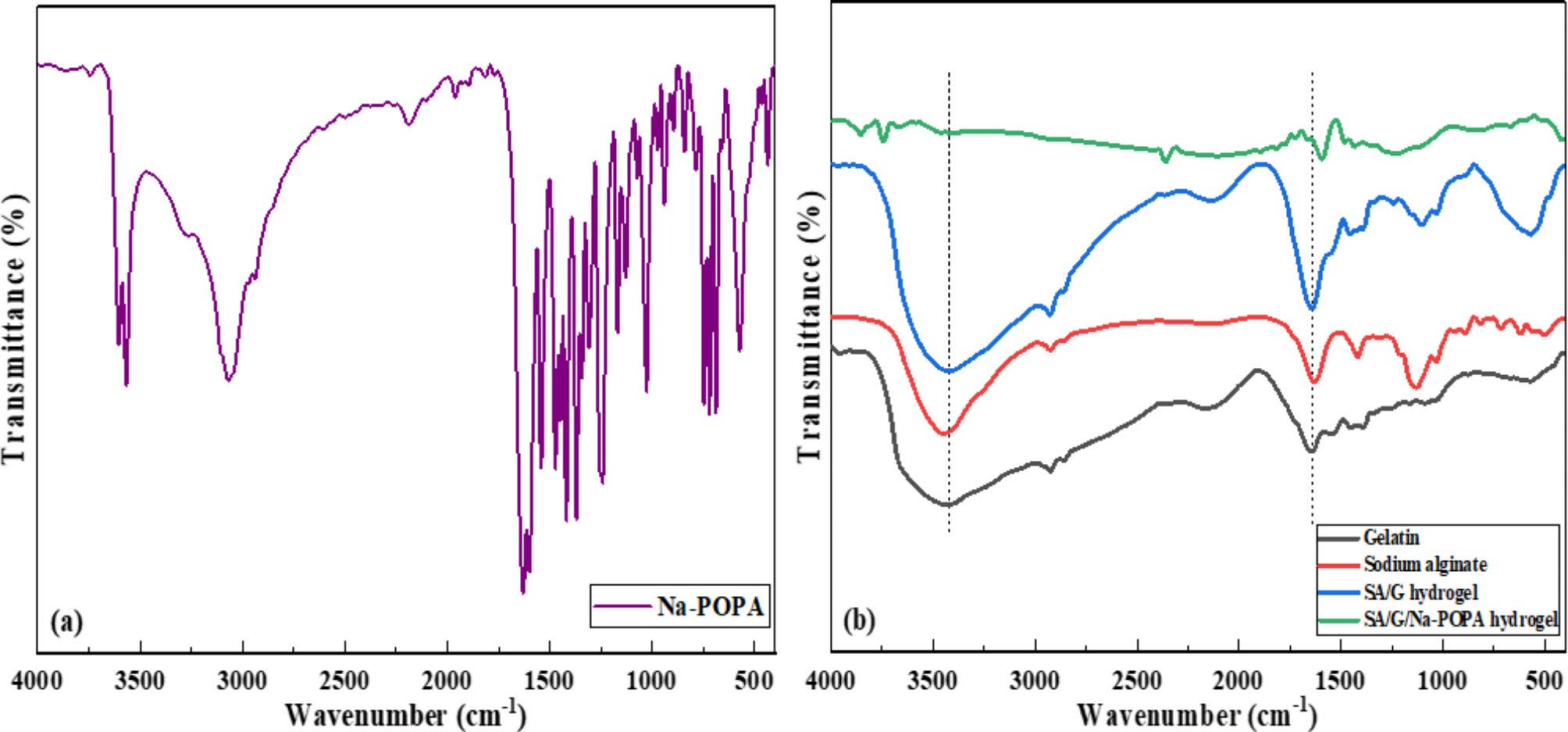
FTIR of (**a**) sodium 2-(2-(3-phenyl-1, 2, 4-oxadiazol-5-yl) phenoxy) acetate and (**b**) gelatin, sodium alginate, SA/G hydrogel, and SA/G/Na-POPA hydrogel.

The IR spectra of Na-POPA, sodium alginate (SA), gelatin (G), SA/G hydrogel and SA/G/Na-POPA hydrogel were recorded between the wavenumbers of 4000 cm^− 1^ and 400 cm^− 1^ as shown in Fig. 4. FTIR spectral analysis was performed to validate the chemical structure of the aforementioned samples. Moreover, the interaction among Na-POPA, SA and gelatin chains in the SA/G/Na-POPA hydrogel sample was confirmed by the noticeable shifts in the wavenumber of certain functional groups^22^.

The FTIR spectrum of Na-POPA showed characteristic absorption bands at 3067.54 and 2939.21 cm^− 1^ corresponding to the C-H stretching vibrations. The vibrational bands at 1629.23 cm^− 1^ and 1599.79 cm^− 1^ corresponding to the aromatic C = C and C = N stretching vibrations respectively.

The FTIR spectrum of sodium alginate revealed characteristic absorption peaks at wavenumbers 3453.43, 2929.80, 1632.46, and 1130.76 cm^− 1^. The peak observed at 3453.43 cm^− 1^ is indicative of the stretching vibration of the -OH group. Additionally, the peak observed at 2929.80 cm^− 1^ corresponds to the C-H stretching vibration, while the peak seen at 1632.46 cm^− 1^ corresponds to the C = O stretching vibration. Lastly, the peak observed at 1130.76 cm^− 1^ can be attributed to the stretching vibration of the carbon-oxygen (C-O) bond^22^.

**Fig. 5:**
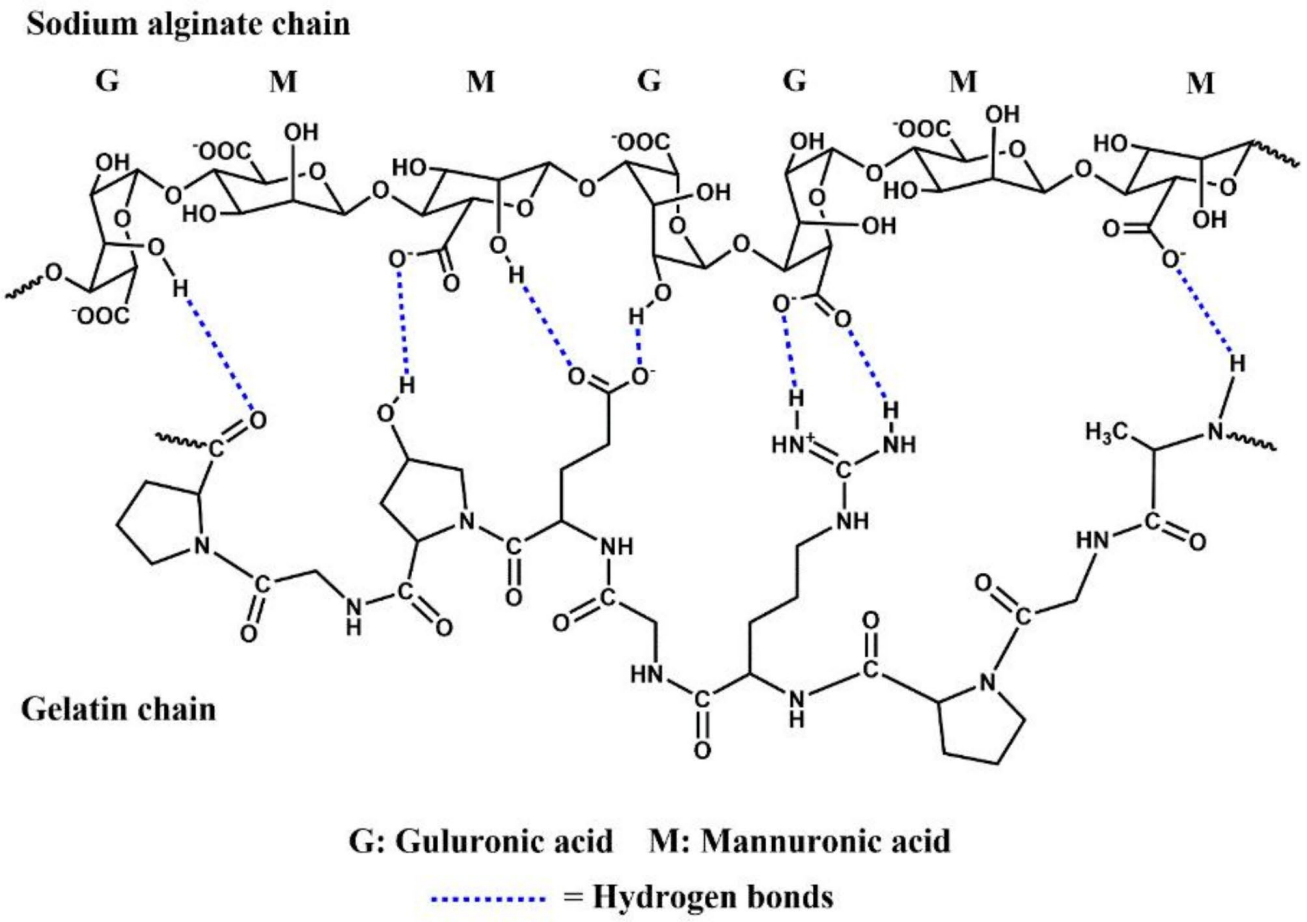
Illustration of the possible interactions between the SA and gelatin chains.

The FTIR spectrum of gelatin exhibited distinct absorption peaks at wavenumbers 3423.38, 2926.06 and 1645.20 cm^− 1^. The prominent peak observed at 3423.38 cm^− 1^ is an indicative of the stretching vibration of the N-H bond of the amide groups, while the peak observed at 2926.06 cm^− 1^ corresponded to the C-H stretching vibration. The presence of amide groups was confirmed by the peak observed at 1645.20 cm^− 1^ which corresponds to the carbonyl group (C = O) stretching vibration of amides. Based on the assigned function groups from FTIR spectra, the intermolecular forces of interaction with the hydrogel matrix are mainly hydrogen bond as the proposed sketch in Fig. 5.

**Fig. 6:**
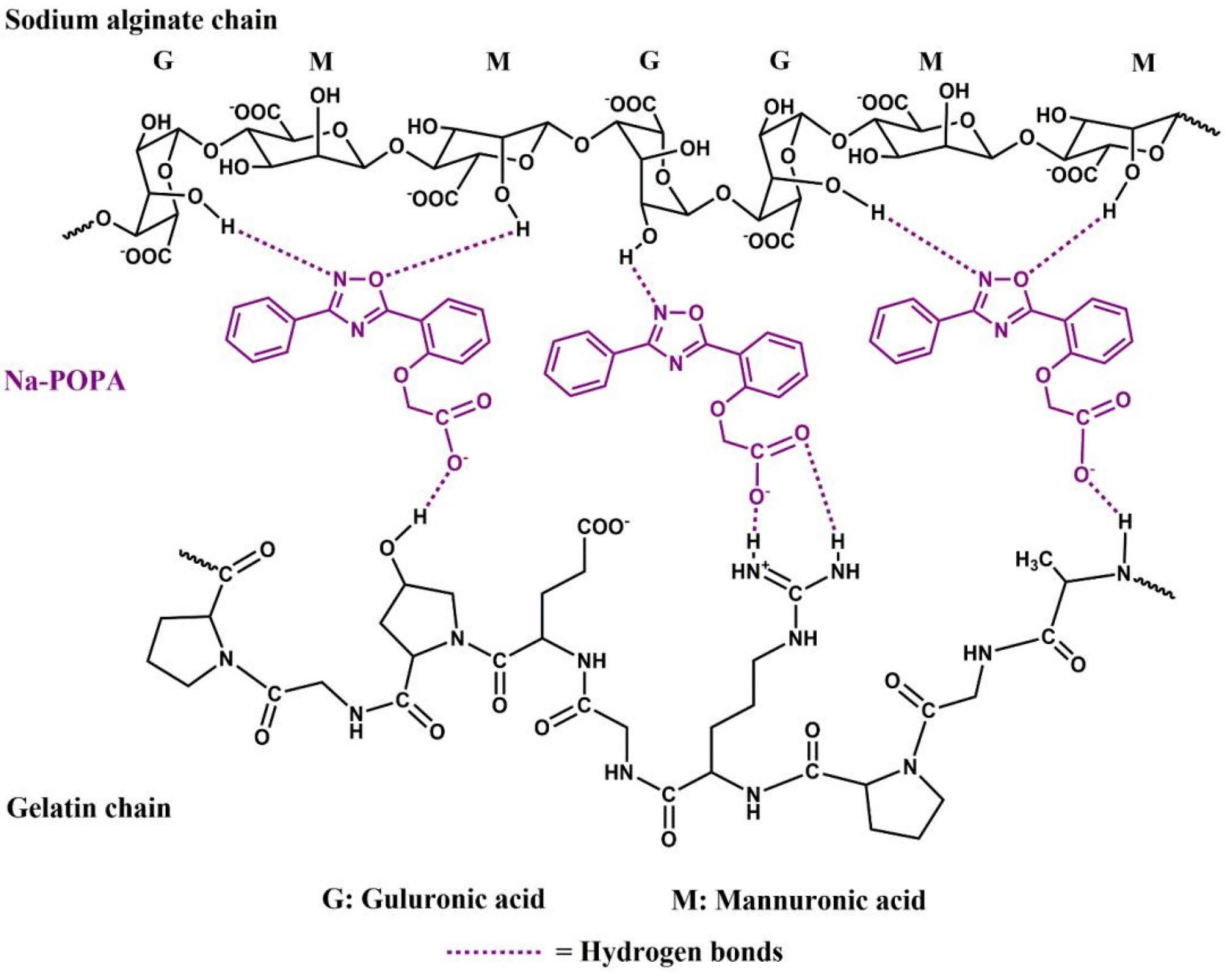
Illustration of the possible hydrogen bond interactions between Na-POPA and the SA and gelatin chains.

Meanwhile, the FTIR spectrum of the SA/G hydrogel showed a highly characteristic broad band at 3415.30 cm^− 1^ which can be attributed to the O-H bond stretching vibration as well as the N-H stretching vibration of amides. The severe broadness of the band suggests the association between the sodium alginate and gelatin chains through hydrogen bonds. Another significant peak was observed at 1642.01 cm^− 1^ which is characteristic for the C = O stretching vibration of amides. The peaks corresponding to the N-H and C = O stretching vibrations of the amide group shifted from 3423.38 to 1645.20 cm^− 1^ in gelatin to 3415.30 and 1642.01 cm^− 1^ in the SA/G hydrogel, respectively. These changes suggest that the negatively charged COO^−^ groups of sodium alginate are associated with the positively charged = NH_2_^+^ groups of gelatin in the SA/G hydrogelas suggested in Fig. 6^22,37,38^.

The presence of Na-POPA in the SA/G/Na-POPA hydrogel was proven by comparing the IR spectra of the SA/G hydrogel with that of the SA/G/Na-POPA hydrogel. It was noticed that the O-H stretching vibration in SA/G/Na-POPA hydrogel shifted to a higher wavenumber (3743.81 cm^− 1^) and became remarkably sharper. This implies a decrease in the intermolecular interaction between sodium alginate and gelatin in SA/G/Na-POPA hydrogel. This decrease is caused by the interaction of Na-POPA with both the gelatin and sodium alginate chains due to its polar nature. Also, the appearance of a peak at 1592.95 cm^− 1^ in SA/G/Na-POPA hydrogel’s IR spectra can be assigned to the C = N stretching vibration of Na-POPA. These results attest to the entrapment of the compound inside the hydrogel successfully^21^.

### Scanning electron microscope (SEM)

**Fig. 7:**
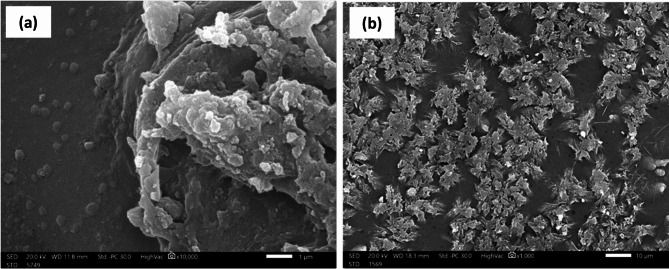
SEM micrograph of (**a**) SA/G hydrogel, and (**b**) SA/G/Na-POPA hydrogel.

The change in the surface morphology due to the integration of Na-POPA inside the SA/G hydrogel matrix was characterized using SEM, as shown in Fig. 7. SA/G hydrogel appeared as water-soaked plain gelatin-alginate layers. The particle size was found to be in micrometers scale, which is in agreement with the common particle size of the hydrogel^15,19^. The self-assembly structure of the hydrogel can be attributed to the extensive intermolecular hydrogen bonding (I.M.B.) between H atoms and either N or O atoms in the gelatin and alginate polymeric chains^15,19^.

The SA/G/Na-POPA hydrogel showed uniform particle size in scale of 10 μm. The larger scale of particle size reflects the entrapment of the compound in the pores of the hydrogel at the expense of breaking down of some I.M.B. Each aggregate represents a self-assembled unit of the compound loaded on the hydrogel^15^.

### X-ray diffraction (XRD) analysis

**Fig. 8:**
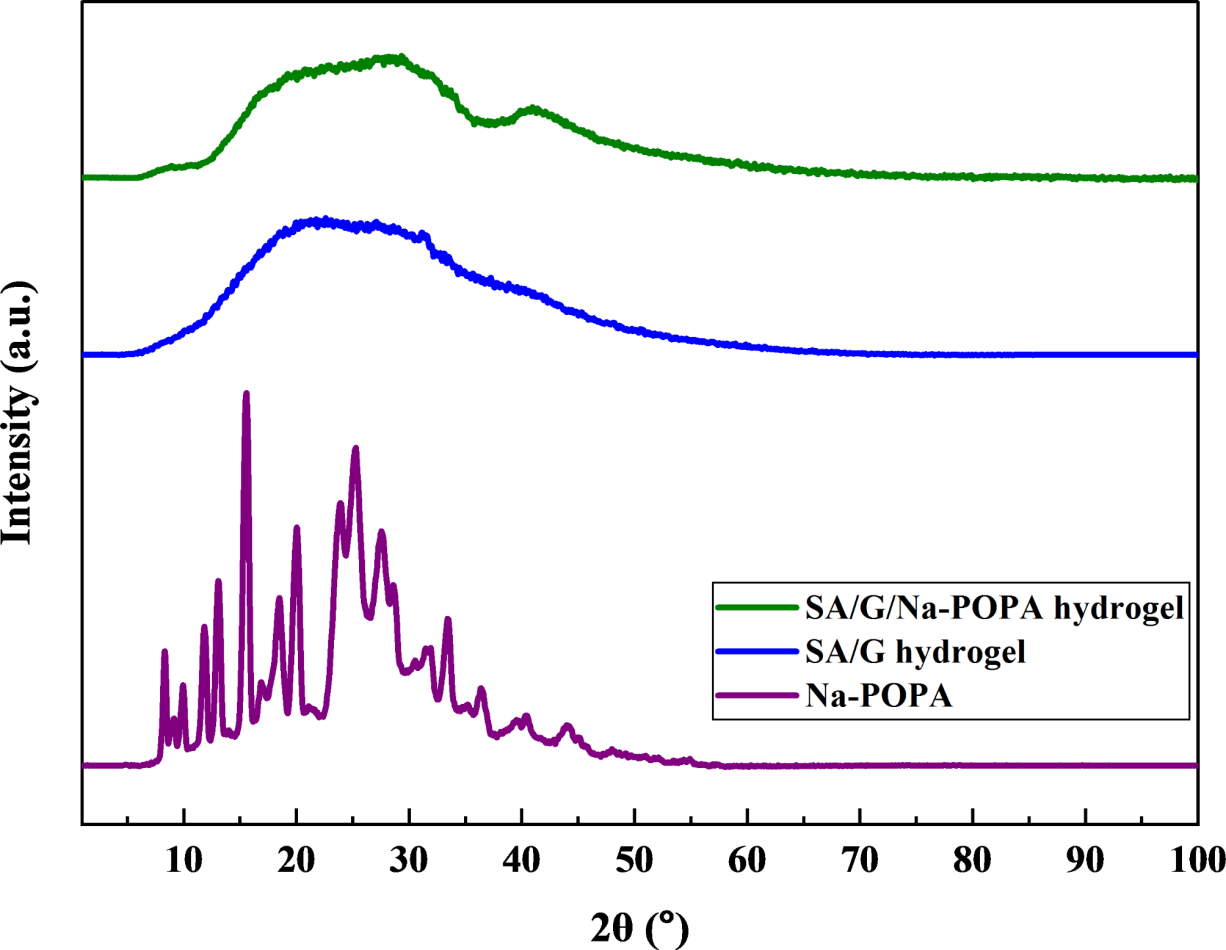
pXRD of Na-POPA, SA/G hydrogel and SA/G/Na-POPA hydrogel.

Figure 8 depicts the X-ray diffraction (XRD) patterns of Na-POPA, SA/G hydrogel, as well as the SA/G/Na-POPA hydrogel. Na-POPA’s diffraction pattern exhibited several sharp diffraction peaks between 2-theta diffraction angle 10° and 40°, which suggests that the compound exists in a well-organized crystalline structure. On the contrary, the SA/G hydrogel and the SA/G/Na-POPA hydrogel display broad, diffuse peaks, indicating they are mostly amorphous and that they lack long-range order, which is often the case in hydrogel matrices^21,22^.

On comparing the pXRD patterns of the SA/G hydrogel and the SA/G/Na-POPA hydrogel, a few subtle differences in the peak positions and intensities may be noticed. These changes imply the incorporation and interaction of the compound within the hydrogel matrix potentially enhancing the functional properties of the hydrogel. Besides, the absence of the distinct crystalline peaks of Na-POPA in the SA/G/Na-POPA hydrogel pattern suggests that the compound is well dispersed within the hydrogel network polymeric matrix^15^.

### Antimicrobial activity

#### Well diffusion method

Antimicrobial activity for Na-POPA, SA/G hydrogel, and SA/G/Na-POPA hydrogel was firstly screened against Gram-positive bacteria; *Staphylococcus aureus* (ATCC 25923) and Gram-negative bacteria *Escherichia coli* (ATCC 25922) by well diffusion assay for determination of the zone of inhibition^39^.Na-POPA, SA/G hydrogel, and SA/G/Na-POPA hydrogel were then tested for determination of their minimum inhibitory concentration by microdilution assay against Gram-positive bacteria *Staphylococcus aureus* (ATCC 25923) and *MRSA* (ATCC 43300) and Gram-negative bacteria *Escherichia coli* (ATCC 25922) and *Klebsiella pneumonia* (ATCC 700603) according to the CLSI 2018 guidelines^39^.

#### Minimum inhibitory concentration (MIC)

Results of MIC determination on both Gram-positive, Gram-negative and MRSA revealed that Na-POPA, SA/G hydrogel and SA/G/Na-POPA hydrogel displayed the same antibacterial profile. They are > two folds more active against MRSA than AMOX standard antibiotic. Similarly, they are > two folds more active against *Klebsiella pneumonia* than AMOX. Additionally, moderate antibacterial effects are exhibited by these samples against *Staphylococcus aureus* and *Escherichia coli*. (Table 1).

**Table 1:** MIC (µg/ml) of Na-POPA, SA/G hydrogel and SA/G/Na-POPA hydrogel compared to AMOX.

Compound	Staphylococcus aureus (ATCC 25923)	MRSA (ATCC 43300)	Escherichia coli (ATCC 25922)	Klebsiella pneumoniae (ATCC 700603)
Na-POPA	250	250	250	250
SA/G hydrogel	250	250	250	250
SA/G/Na-POPA hydrogel	250	250	250	250
Amoxicillin	≤ 7.8125	> 500	62.5	> 500

### Cytotoxicity assay

**Fig. 9:**
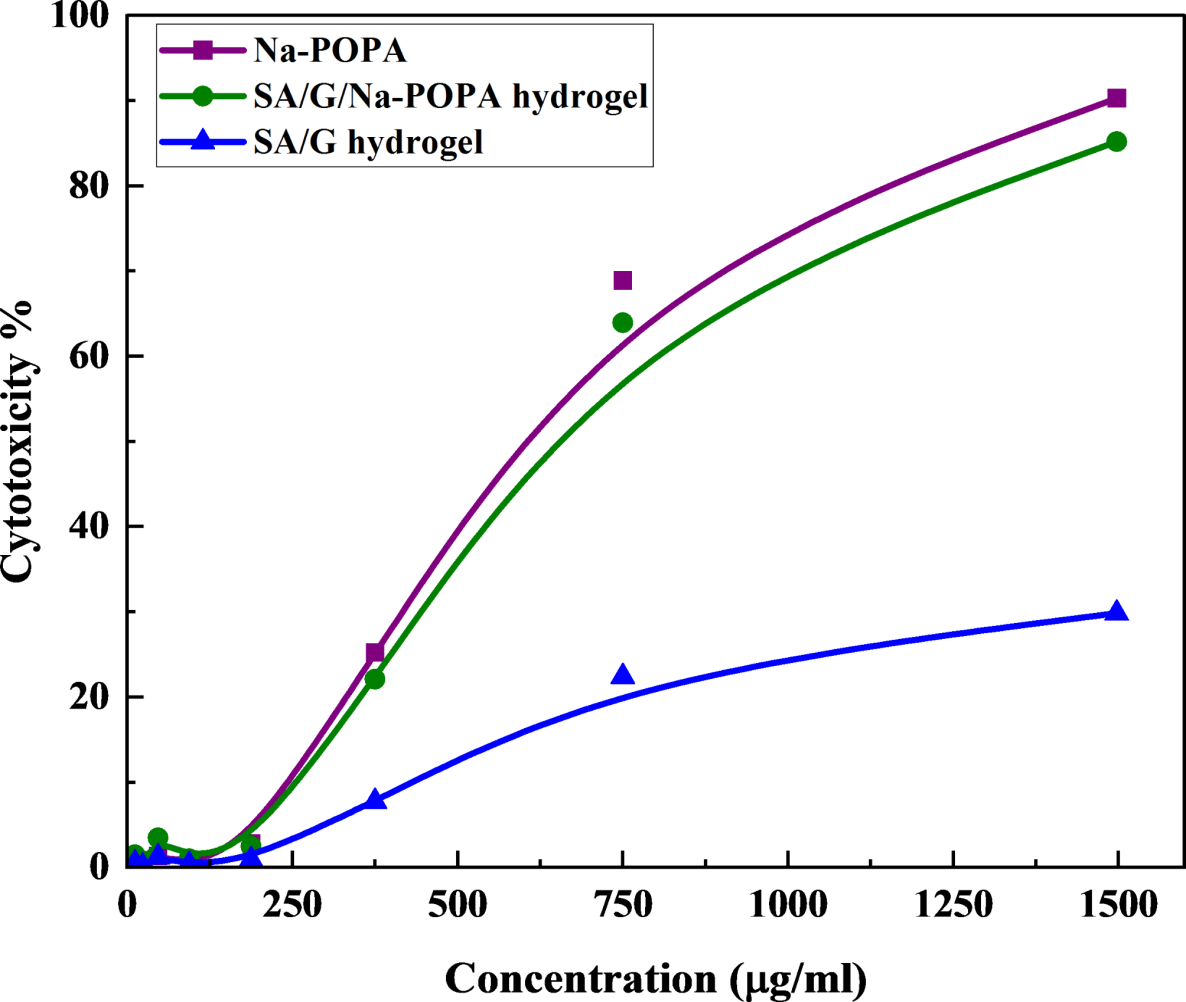
Cytotoxicity % of Na-POPA, SA/G hydrogel and SA/G/Na-POPA hydrogel.

Figure 9 illustrates the comparative cytotoxic effects of Na-POPA, SA/G hydrogel, and SA/G/Na-POPA hydrogel against the normal human fetal lung fibroblast cell lines (MRC-5).

SA/G hydrogel showed minimal cytotoxicity, which was expected due to the biocompatible nature of both gelatin and sodium alginate. On the other hand, the Na-POPA sample exhibited the highest cytotoxicity, with an IC_50_ value of 534.45 µg/mL. The SA/G/Na-POPA hydrogel showed an IC_50_ of 553.22 µg/mL. The increase in the IC_50_ value indicates that loading the Na-POPA onto the hydrogel resulted in a notable decrease in its cytotoxicity compared to pure Na-POPA. These findings imply that the hydrogel formulation has the ability to mitigate the cytotoxic effect of the heterocyclic compound, making it more compatible with the cells in human body^40,41^.

### Kinetic studies

#### In vitro release study

Using spectrophotometry, the release of Na-POPA from the hydrogel was examined in vitro in different pH buffer solutions. Two buffer solutions with pH 1.2 and pH 6.8 were chosen to simulate the pH of biological fluids in stomach and colon respectively in the human body. In this instance, 25 mL of the intended buffer was mixed with 0.05 g of the SA/G/Na-POPA hydrogel at 37℃ ±0.5℃ to simulate the human body temperature, as per the experimental section’s instructions. Using a UV-Visible spectrophotometer, the absorbance change in the supernatant at λ_max_. 245 nm was used to analyze to determine the amount of the drug release.

**Fig. 10:**
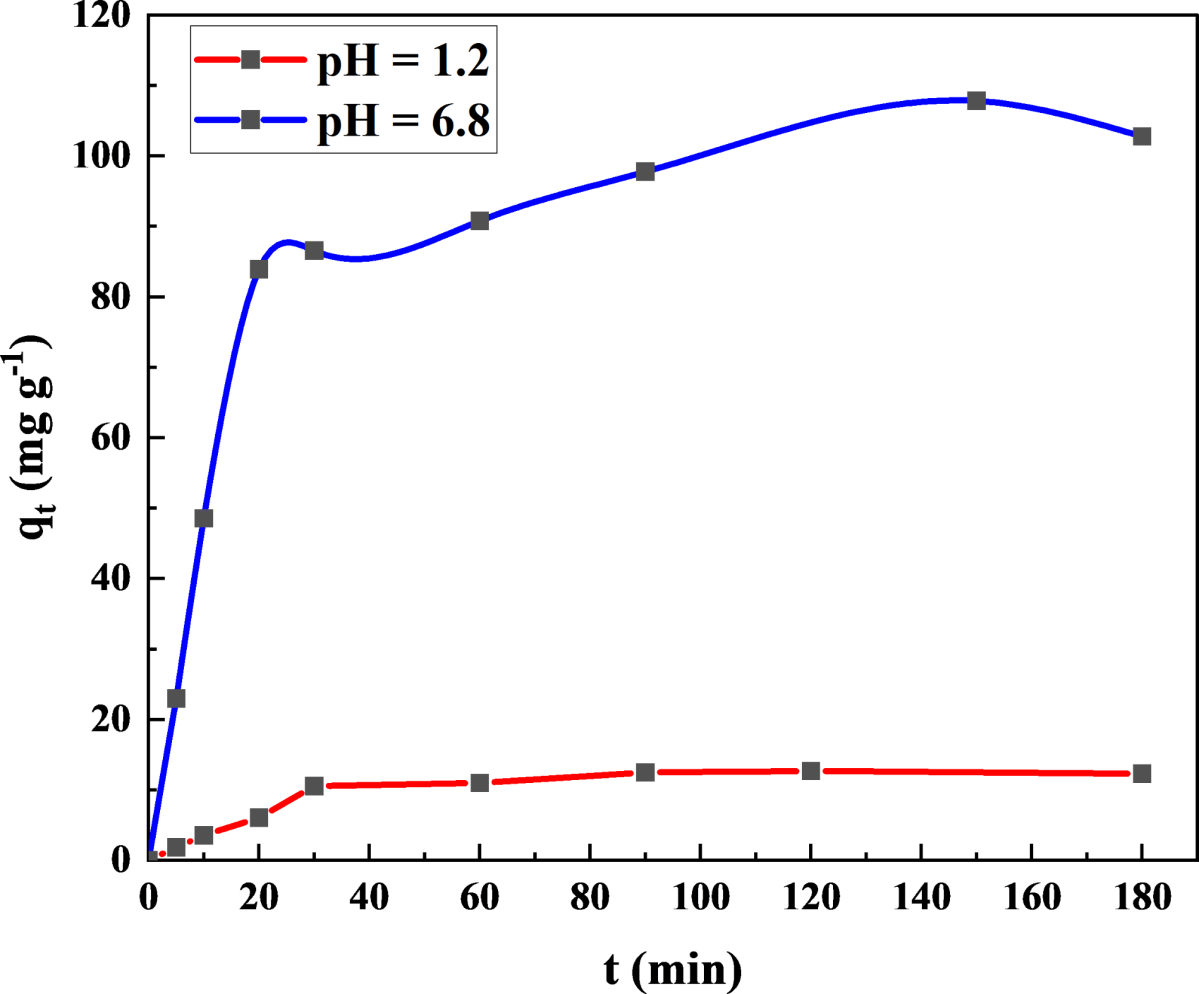
Cumulative in vitro release of Na-POPA from the hydrogel at pH 1.2 and pH 6.8.

The data displayed in Fig. 10 demonstrated that the release of Na-POPA achieved a stable plateau after 30 min at pH 1.2, whereas the delivery was still ongoing at pH 6.8. After 30 min., the percentage release is 85.67% and 80.25%, for pH values 1.2 and 6.8 respectively.

None of the samples showed a release burst, indicating that the compound is evenly dispersed throughout the matrix and is not just gathered on the carrier surface. The release kinetic is dependent on the pH of the aqueous environment solution. The amount released at pH 6.8 was noticeably more than the amount released at pH 1.2 according to the release data. This may be attributed to the presence of substantial electrostatic repulsion between the negatively charged groups in sodium alginate and gelatin at pH 6.8, which led to the quick relaxation of the polymeric chains and the induction of Na-POPA release, whereas at pH 1.2, protonation of some amino groups a in sodium alginate and gelatin and Na-POPA retarded release^35^.

#### Modeling of Na-POPA release

To understand the kinetic mechanism involved in the hydrogel’s release of Na-POPA, a variety of empirical formulas of kinetic models are used based on the hydrogel’s nature and the behavior of the compound’s release from the hydrogel at different pH values (1.2 and 6.8). Pseudo-first-order, pseudo-second-order, zero-order, Elovich, Huguchi, and Ritger-Peppas and Korsmeyer models are among the empirical equations that were used for fitting the release data^42,43^. The Elovich model explains a release process through bulk surface diffusion, the Korsmeyer-Peppas model posits that the release happens through diffusion from surfaces.

**Fig. 11:**
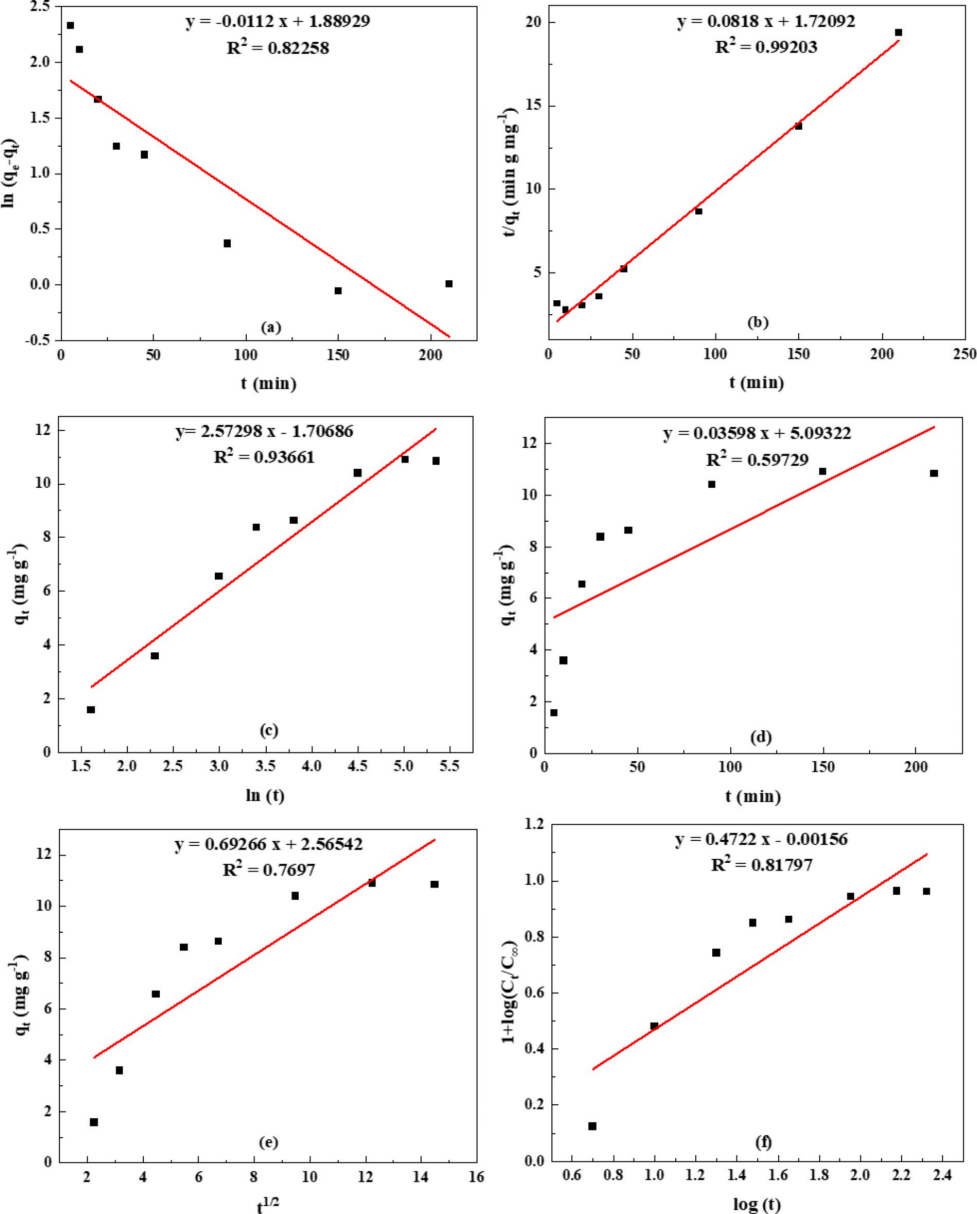
Fitting of the data for Na-POPA release from SA/G hydrogel into (**a**) pseudo-first-order, (**b**) pseudo-second-order, (**c**) Elovich, (**d)** zero-order, (**e**) Huguchi and (**f**) Ritger-Peppas and Korsmeyer-Peppas at pH 1.2.

**Fig. 12:**
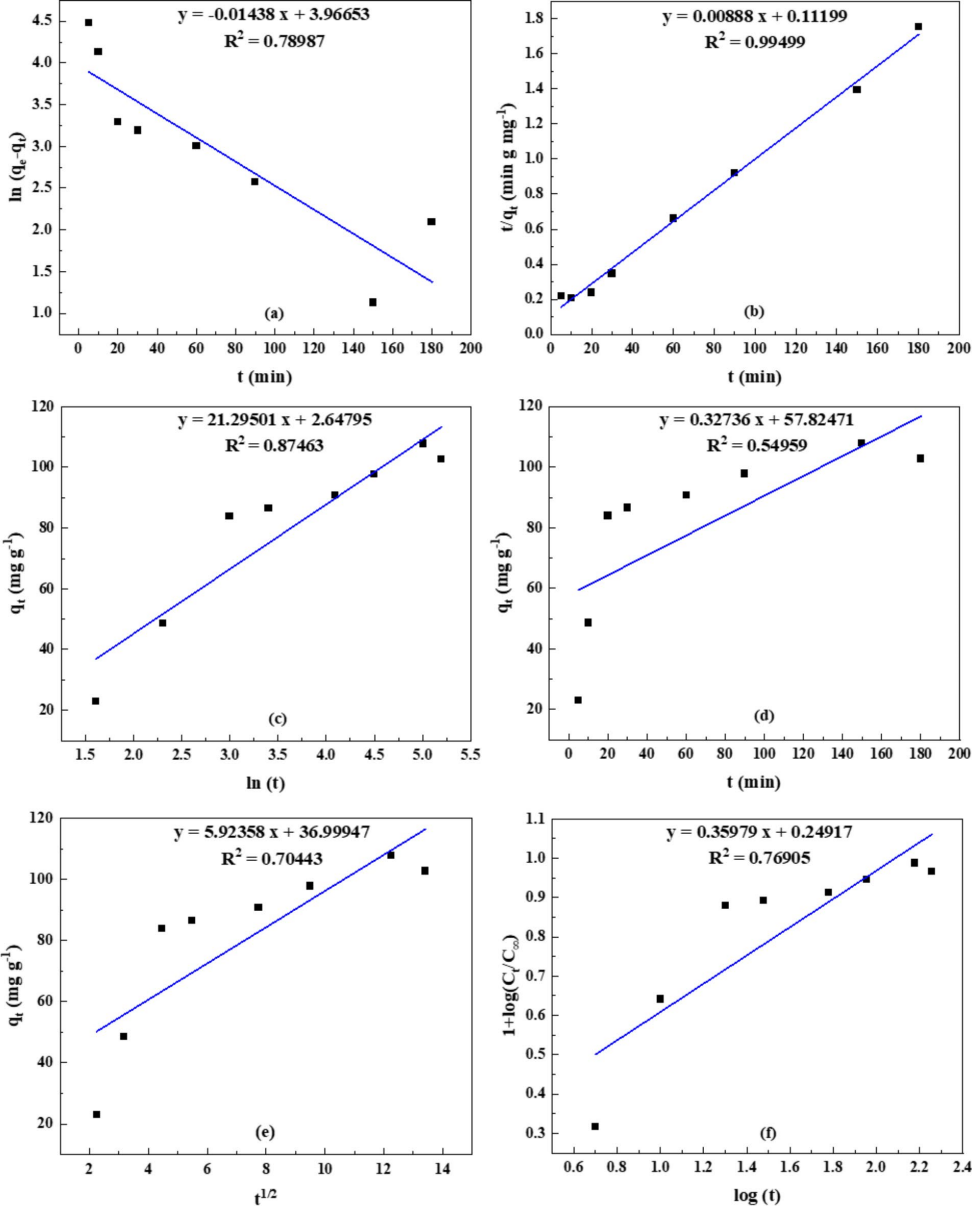
Fitting of the data for Na-POPA release from SA/G hydrogel into (**a**) pseudo-first-order, (**b**) pseudo-second-order, (**c**) Elovich, (**d**) zero-order, (**e**) Huguchi and (**f**) Ritger-Peppas and Korsmeyer-Peppas at pH 6.8.

The kinetic models suggest that the first-order model mostly represents the release behavior by dissolving. Among these kinetic models, it was found that the release kinetics process of Na-POPA from the hydrogel at pH 1.2 and 6.8 were well obeyed by the pseudo-second-order model as depicted in Figs. 11 and 12. The R^2^ values were near unity (0.980–0.995) on applying the pseudo-second-order kinetic Eqs. 1, 2.

1 $$\:\:\:\:\:\:\:\:\:\:\:\:\:\:\text{(}\frac{\text{t}}{{\text{q}}_{\text{t}}}\text{)=\:}\frac{\text{1}}{{\text{k}}_{\text{2\:\:\:}}{\text{q}}_{\text{e}}^{\text{2}}}\text{+\:}\frac{\text{1}}{{\text{q}}_{\text{e}}}\left(\text{t}\right)\:$$

2 $$\:\text{I}\text{nitial\:}\text{release\:rate\:(mg\:g}\text{-1}\text{min}\text{-1}\text{)\:}\text{=\:h\:=}\frac{\text{d}\text{\:}{\text{q}}_{\text{t=0}}}{\text{d}\text{\:}\text{t}}\text{=\:}{\text{k}}_{\text{2}}{\text{\:}\text{q}}_{\text{e}}^{\text{2}}$$

Where *k*_*2*_ (mg g^− 1^min^− 1^) is the rate constant of second-order release. The values *k*_2_, *q*_e_, *h*, and correlation coefficients *R*^*2*^ were calculated from the plot of *t/q* versus *t*, and h (the reaction rate at t = 0) was calculated from the slope of *q*_*t*_ versus time.

The kinetic parameters of the pseudo-second-order model are gathered in Table 2. The data indicated that the initial rate was about twice as high when computed using the pseudo-second-order model as opposed to the initial slope. Moreover, the results demonstrated that after 30 min., the initial rate for pH 6.8 is approximately ten times higher than pH 1.2, after which equilibrium is established for 1.2 and the release is still ongoing at pH 6.8. As a result, the rate constant k_2_ obtained is higher at pH 1.2 than pH 6.8. In this kinetic model, the rate-limiting step is the release of the chemisorbed compound from the surface, where the removal of the compound from a hydrogel is due to physicochemical interactions between the aqueous buffer solution and the hydrogel matrix^44,45^.

**Table 2:** Correlation coefficient (R^2^), rate constant (k_2_), initial rate (h, h^/^) and q_e_ values obtained by fitting the release data of Na-POPA at pH 1.2 and 6.8 to the pseudo-second-order kinetic model.

pH	*R* ^2^	Rate constant k_2_ (x10^3^) (g mg^− 1^ min^− 1^)	Initial rate (g mg^− 1^ min.^−1^)		q_e_ (mg g^− 1^)
			h	h^/^	
1.2	0.98	2.74	0.59	0.34	14.70
1.2	0.99	3.88	0.58	0.27	12.22
6.8	0.99	0.52	10.94	7.60	144.92
6.8	0.99	0.71	8.92	4.20	112.35

### Electrochemical analysis

**Fig. 13:**
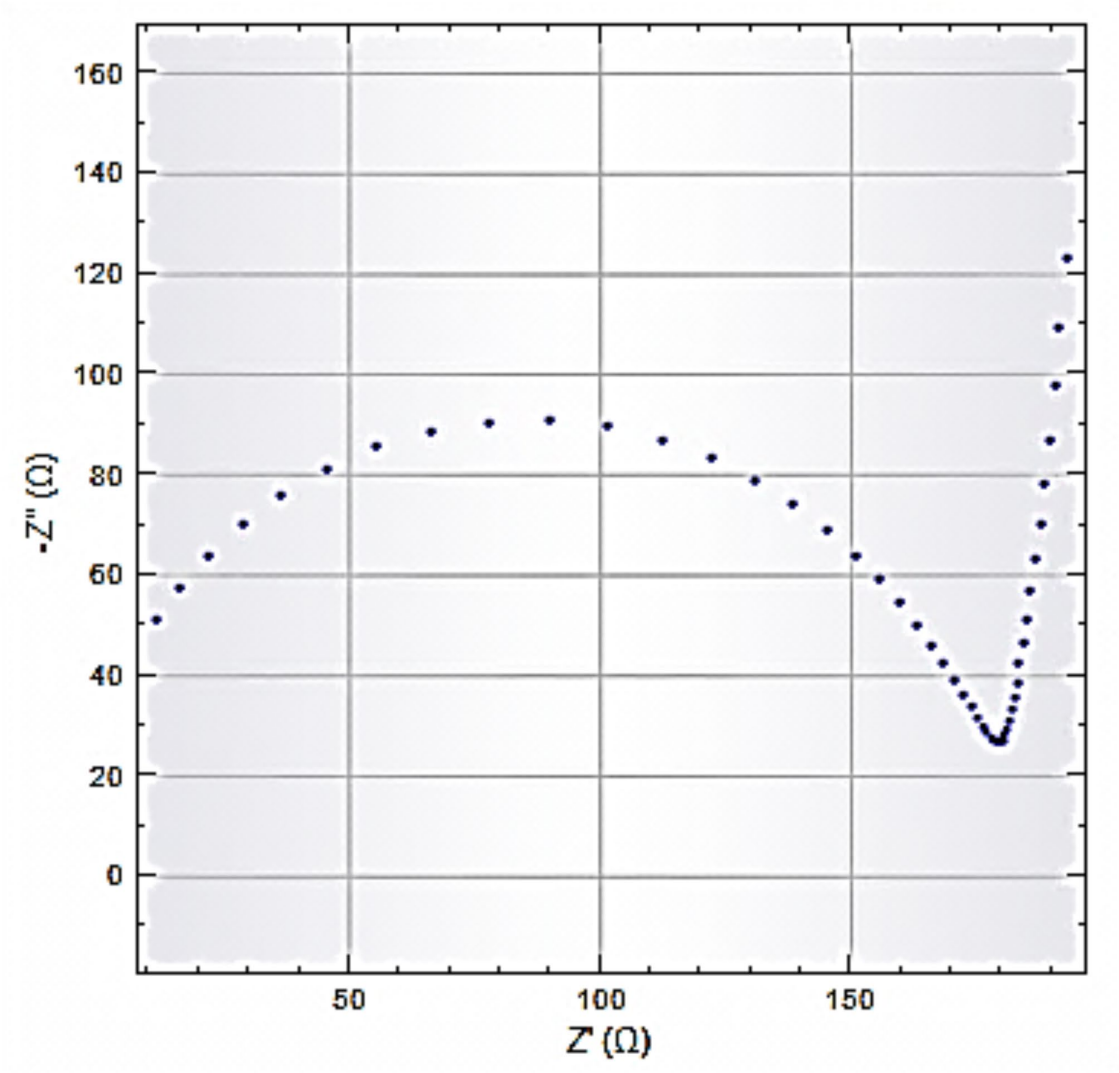
Impedance plot for release of Na- POPA at pH 1.2.

**Fig. 14:**
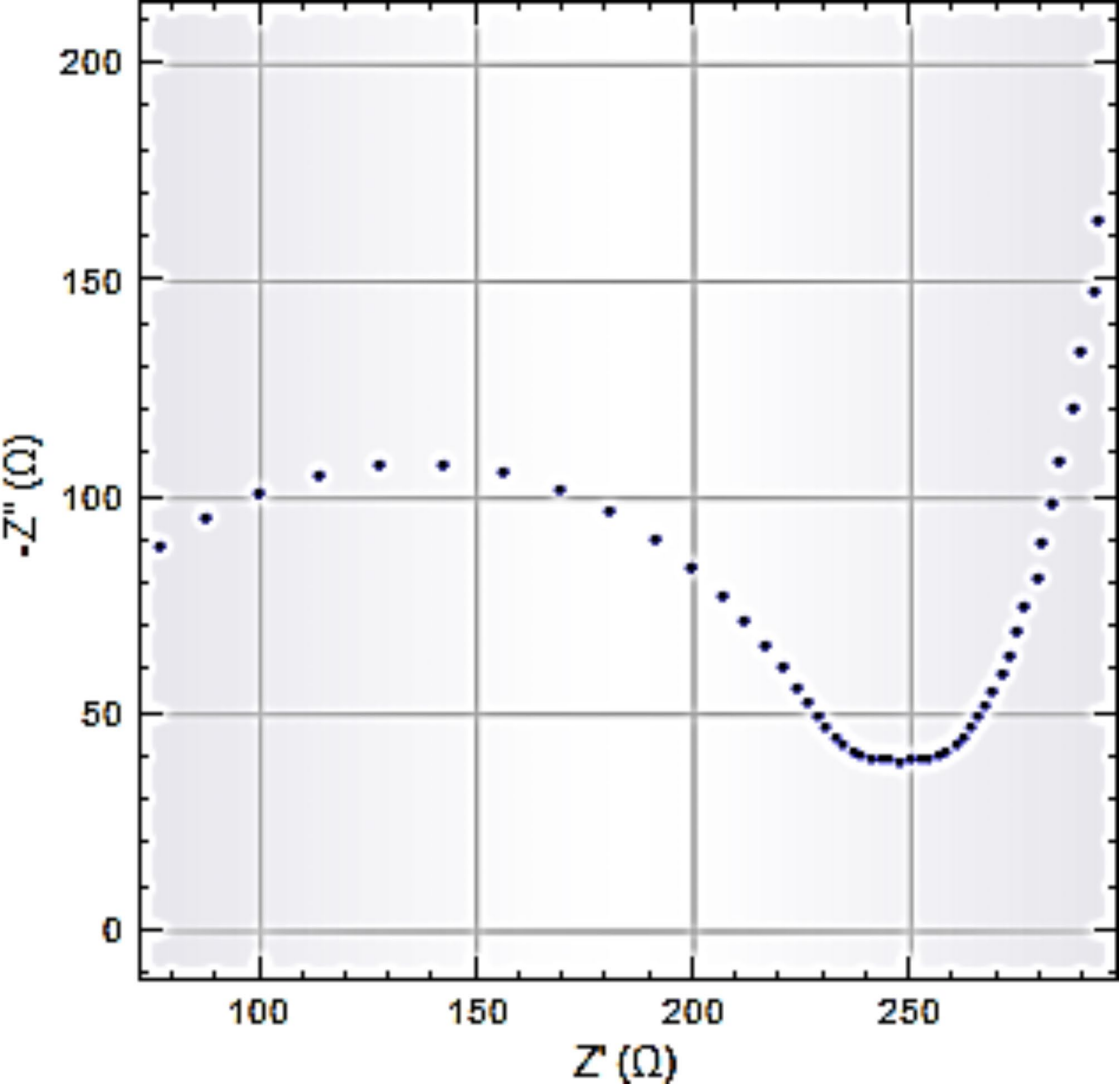
Impedance plot for release Na-POPA at pH 6.8.

**Fig. 15:**
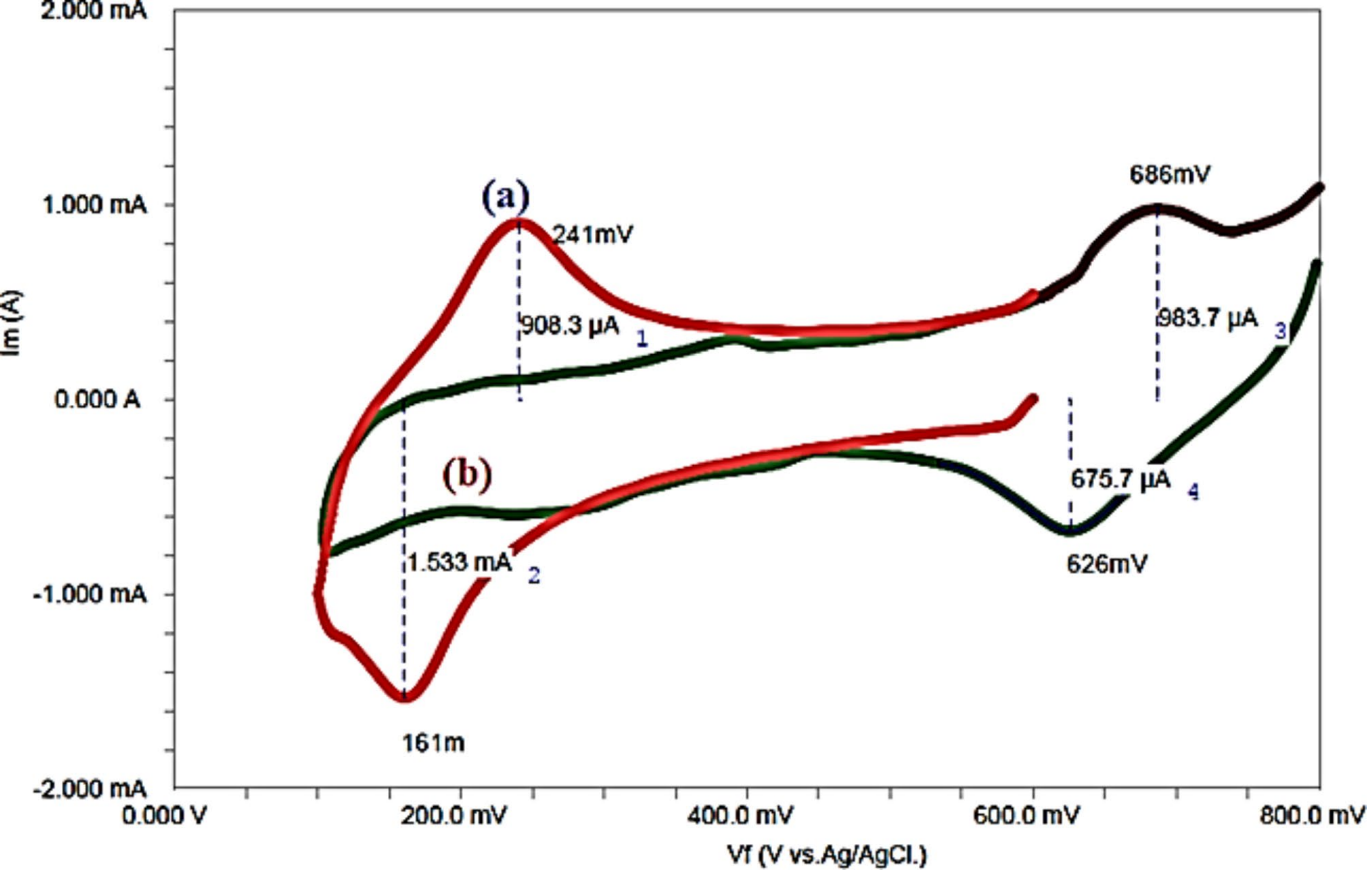
Comparative CV for release Na-POPA: (**a**) pH 1.2 and (**b**) pH 6.8.

The electrochemical tests confirmed Na-POPA release is controlled by the nature of the biodegradable SA/G polymer carrier. In buffer solution, swelling of the hydrogel by water caused expansion of the polymer matrix, which enhanced rupture, degradation and releasing of Na-POPA from the confined inner polymeric matrix. Na-POPA molecules were released *via* bond breaking or desorption. Figures 13, 14 and 15 show the comparative release of Na-POPA at pH 1.2 (gastric) and 6.8 (intestinal), simulating physiological body fluids^21^.

Na-POPA release was pH-dependent with a larger amount released at 6.8 (near the pH of the intestinal system). Na-POPA molecules dissolved more at pH 6.8 and therefore reached the surface of WE, adsorbed and impeded the passage of current^21,41,46^.

In acidic pH 1.2, the negatively charged carboxylate –COO^−^ groups of SA become protonated, became water insoluble and hindered the diffusion of Na-POPA molecules to the surface of WE. The release in this case required a longer time. Buffer solution pH 6.8 (intestinal pH) exhibited more leaching of Na-POPA molecules than gastric pH 1.2.

All these findings confirmed that the SA/G polymeric drug carrier is pH-responsive, where it shrinks at low pH 1.2while effectively swells at high pH 6.8. Moreover, the formulated drug carrier can hold drugs till reaching and targeting the cell uptake.

## Conclusion

In the current study, a pH-responsive physically cross-linked SA/G hydrogel prepared that used in different drug delivery systems. The new 1, 2, 4-oxadiazole derivative prepared and showed adequate antibacterial activity against both Gram-positive and Gram-negative bacteria. The compound was successfully incorporated into the SA/G hydrogel by batch synthesis method. Post-synthesis characterization of the SA/G and SA/G/Na-POPA hydrogels using FTIR, SEM, and pXRD confirmed the chemical structure of the new heterocyclic compound. Also the results of characterization analysis confirmed the interaction by molecules Na-POPA and the cross-linked hydrogel chains. The heterocyclic compound entrapped inside the hydrogel matrix. Evaluation of the cytotoxicity levels of both pure Na-POPA and loaded Na-POPA revealed that the hydrogel reduced the cytotoxicity of the compound, thus improved its biocompatibility. The release profiles of Na-POPA from the hydrogel were sustained, with greater cumulative release of Na-POPA at pH 6.8 than at pH 1.2. The pseudo-second-order model was found to be the best kinetic model fitted the release data. Further studies are to be carried out to test for the oral tolerability of the hydrogel composite. Additionally, in vivo studies are required to confirm the therapeutic potential of the present delivery system for the pro-drug Na-POPA.

## Electronic supplementary material

Below is the link to the electronic supplementary material.

Supplementary Material 1

## Data Availability

“All data generated or analysed during this study are included in this published article”.
