# Supplementary material for: Preparation and kinetic studies of a new antibacterial sodium alginate gelatin hydrogel composite
Source: Sci Rep. 2024 Nov 25;14:29206. doi: 10.1038/s41598-024-80453-8 (PMC11589701; doi:10.1038/s41598-024-80453-8)
Supplement: Supplementary file 1 — Supplementary Material 1 [file 41598_2024_80453_MOESM1_ESM.pdf]

Supporting Information

Preparation and kinetic studies of a new antibacterial sodium alginate gelatin hydrogel composite

Reem A. ElTatawy [a], Amel M Ismail [a], Mohammed Salah Ayoup [b,a], Magda M. F. Ismail [c], Howida Abouel Fetouh [a*]

[a] Department of Chemistry, Faculty of Science, Alexandria University, Alexandria, 21321, Egypt

[b] Department of Chemistry, College of Science, King Faisal University, Al-Ahsa 31982, Saudi Arabia

[c] Department of Pharmaceutical Medicinal Chemistry, Faculty of Pharmacy (Girls), Al-Azhar University, Cairo 11651, Egypt.

*Howida_fetouh@alexu.edu.eg

Content **Page**

Content	Page
**SI.1.Materials and Equipment**	**S2**
**SI.2.Determination of minimum inhibitory concentration (MIC) by microdilution assay**	**S2**
**SI.3.Cytotoxicity assay**	**S3**
**SI.4.^{1}H NMR, ^{13}C NMR and HRMS spectra**	**S4-S8**

SI.1. Materials and instruments

Gelatin type B (pharmaceutical grade, 250 bloom from bovine bone) was purchased from The Arab Company for Gelatin & Pharmaceutical products, El Amreya, Egypt. Sodium alginate was purchased from DOP Organik Kimya, Turkey.

All reactions were carried out in dried glasswares. NMR spectra were measured using a JEOL JNM-ECA 500 MHz (Japan). The deuterated solvent was used as an internal deuterium lock. ^{13}C NMR spectra were recorded using the UDEFT pulse sequence and broad band proton decoupling at 125 MHz. All chemical shifts (δ) are stated in units of parts per million (ppm) and presented using TMS as the standard reference point. IR (KBr) ν_{max} (cm^{-1}) data were recorded using Bruker tensor 37 FT-IR (Germany). The HRMS was recorded on LC/Q-TOF, 6530 (Agilent Technologies, Santa Clara, CA, USA) at Faculty of Pharmacy, Fayoum University.

The surface morphology of the hydrogels was examined by a JEOL JSM-5300 scanning electron microscope (Japan). XRD spectra were measured using Bruker X-ray powder diffraction-XRD-D2 Phaser (Germany). The release profiles of Na-POPA from the SA/G/Na-POPA hydrogel were studied using PG T80+ UV-Vis double beam spectrophotometer (United Kingdom).

SI.2. Determination of minimum inhibitory concentration (MIC) by microdilution assay

Investigation of antimicrobial activity of chemical compounds was performed by microbroth dilution assay for determination of minimum inhibitory concentration (MIC). In summary, 100 µl of Muller-Hinton broth (MHB) (Oxoid® Limited, Basingstoke, UK) were disseminated in 96 multi-well microtiter plates, followed by the addition of 100 µl chemical compound into the first row of the microtiter plate. Then, from the first to the twelfth well, serial dilution was performed. Each well received 7 µl of freshly prepared bacterial suspension ($1.5x10^{8}$cfu/mL). For each bacterial strain, positive and negative controls were carried out. Plates were incubated for 18-24 hours at 37 ˚C, with Amoxicillin 1000 µg/ml serving as reference standard antibiotic. The MIC was estimated as the minimum concentration that demonstrated no detectable bacterial growth.

SI.3.Cytotoxicity assay

The cytotoxic activity of Na-POPA, SA/G hydrogel and SA/G/Na-POPA hydrogel was evaluated against a normal human cell line, human fetal lung fibroblast (MRC-5), using the MTT assay.

Briefly, a 96-well tissue culture plate was seeded with 1×10^5 cells/mL (100 µL/well) and incubated at 37°C for 24 hours to allow the formation of a complete monolayer. Following incubation, the growth medium was removed, and the cell monolayers were washed twice with phosphate-buffered saline (PBS). Two-fold serial dilutions of the test sample were prepared in RPMI medium supplemented with 2% fetal bovine serum (FBS), and 100 µL of each dilution was added to the wells. Three wells were used as negative controls, receiving only maintenance medium (RPMI with 2% FBS). Plates were incubated at 37°C and examined microscopically for any morphological signs of toxicity, such as cell rounding, shrinkage, granulation, or partial/complete loss of the monolayer.

MTT solution (5 mg/mL in PBS, Bio Basic Canada Inc.) was prepared, and 20 µL of MTT solution was added to each well. The plates were placed on a shaking table (150 rpm) for 5 minutes and then incubated at 37°C in a 5% CO_2 atmosphere for 2 hours. The resulting formazan crystals were dissolved in 200 µL dimethyl sulfoxide (DMSO), and the optical density (OD) was measured at 560 nm using a microplate reader.

The half-maximal inhibitory concentration (IC50) values were calculated for each sample. All experiments were performed in triplicate, and the mean optical density values and standard errors were determined.

SI.4.¹H NMR, ¹³C NMR and HRMS spectra

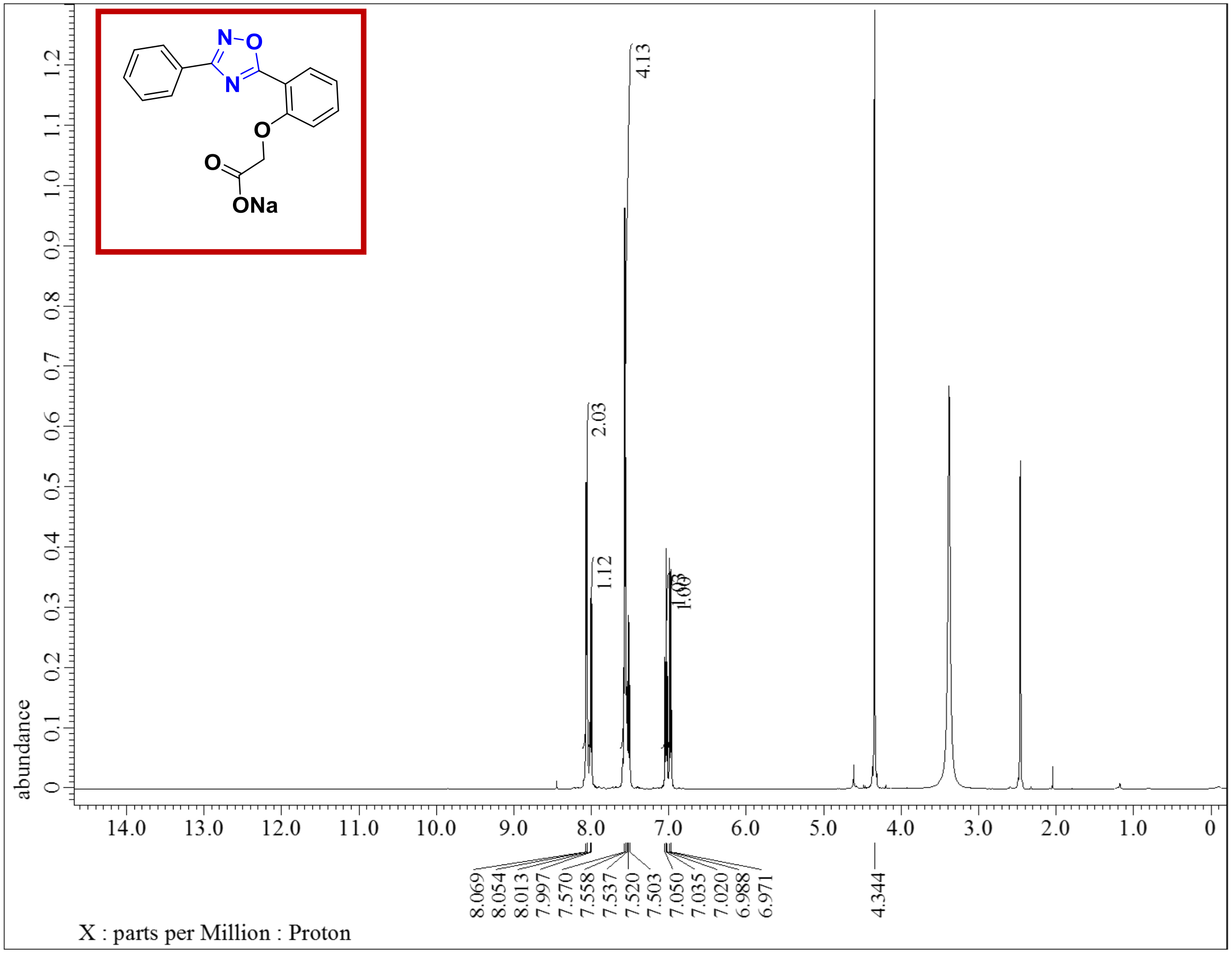

Fig. SI.1 *^{1}H-NMR spectrum (500 MHz, DMSO-d_6) of* ***5****.*

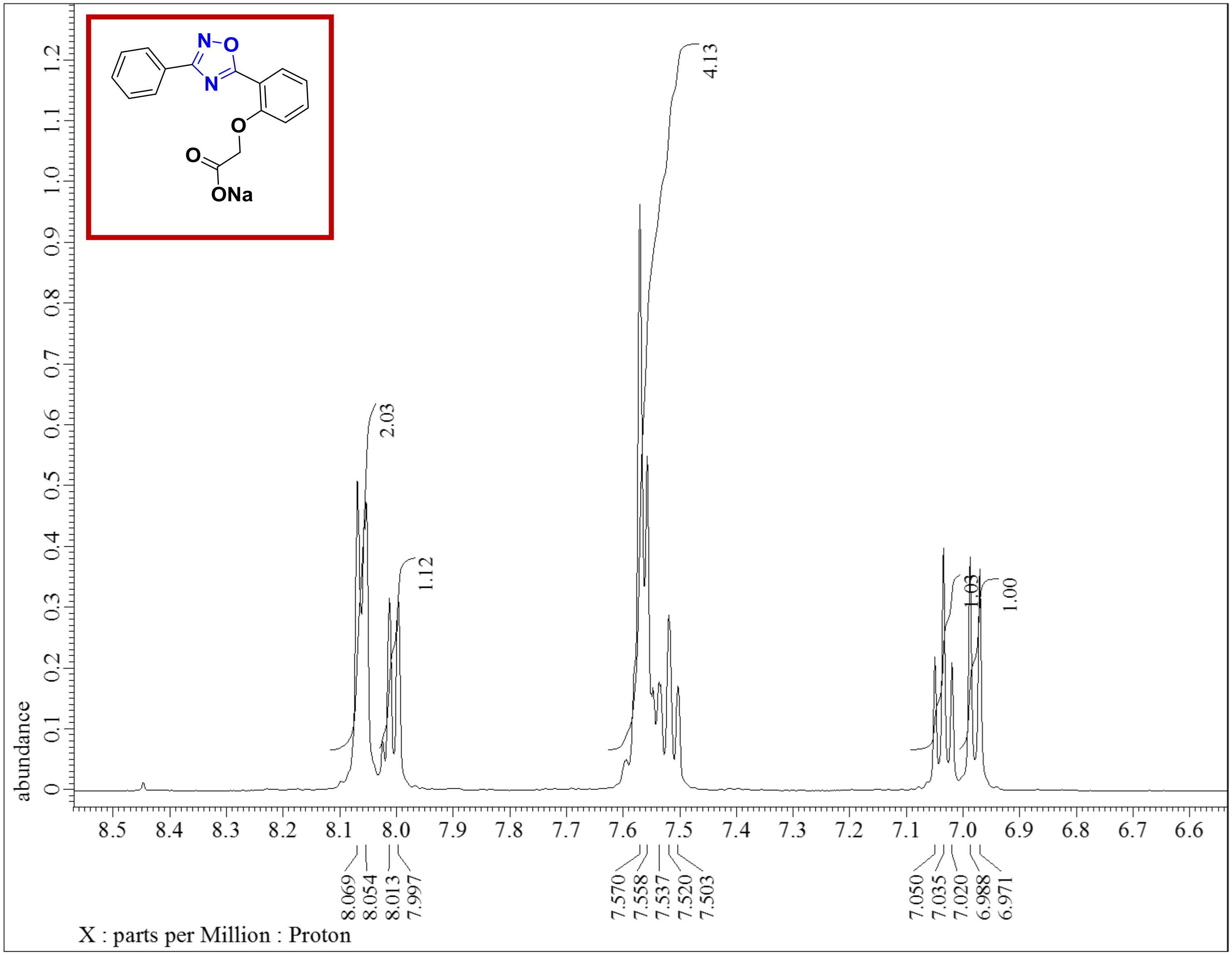

Fig. SI.2 *^{1}H-NMR spectrum (500 MHz, DMSO-d_6) of* ***5****.*

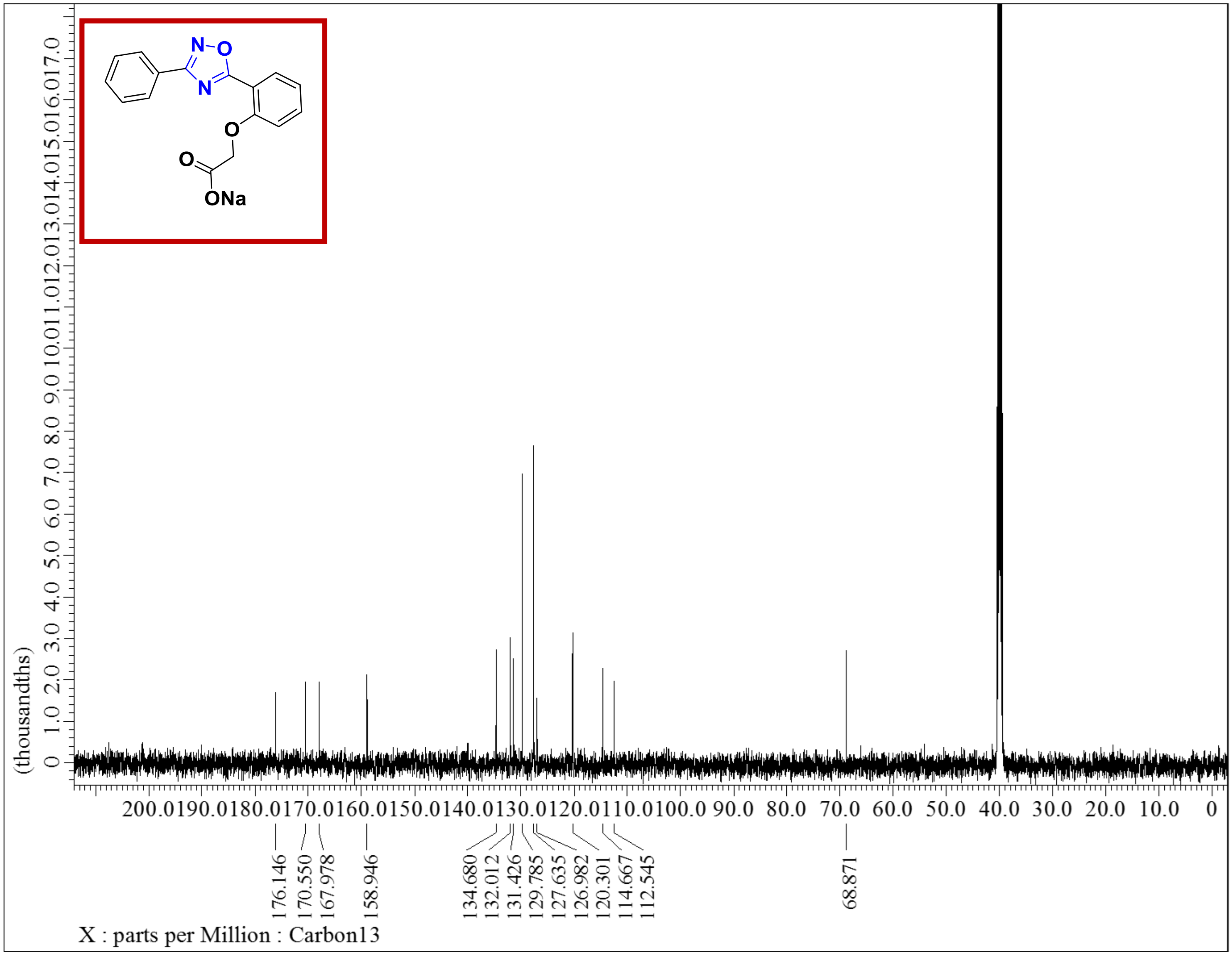

Fig. SI.3 *^{13}C-NMR spectrum (125 MHz, DMSO-d_6) of* ***5****.*

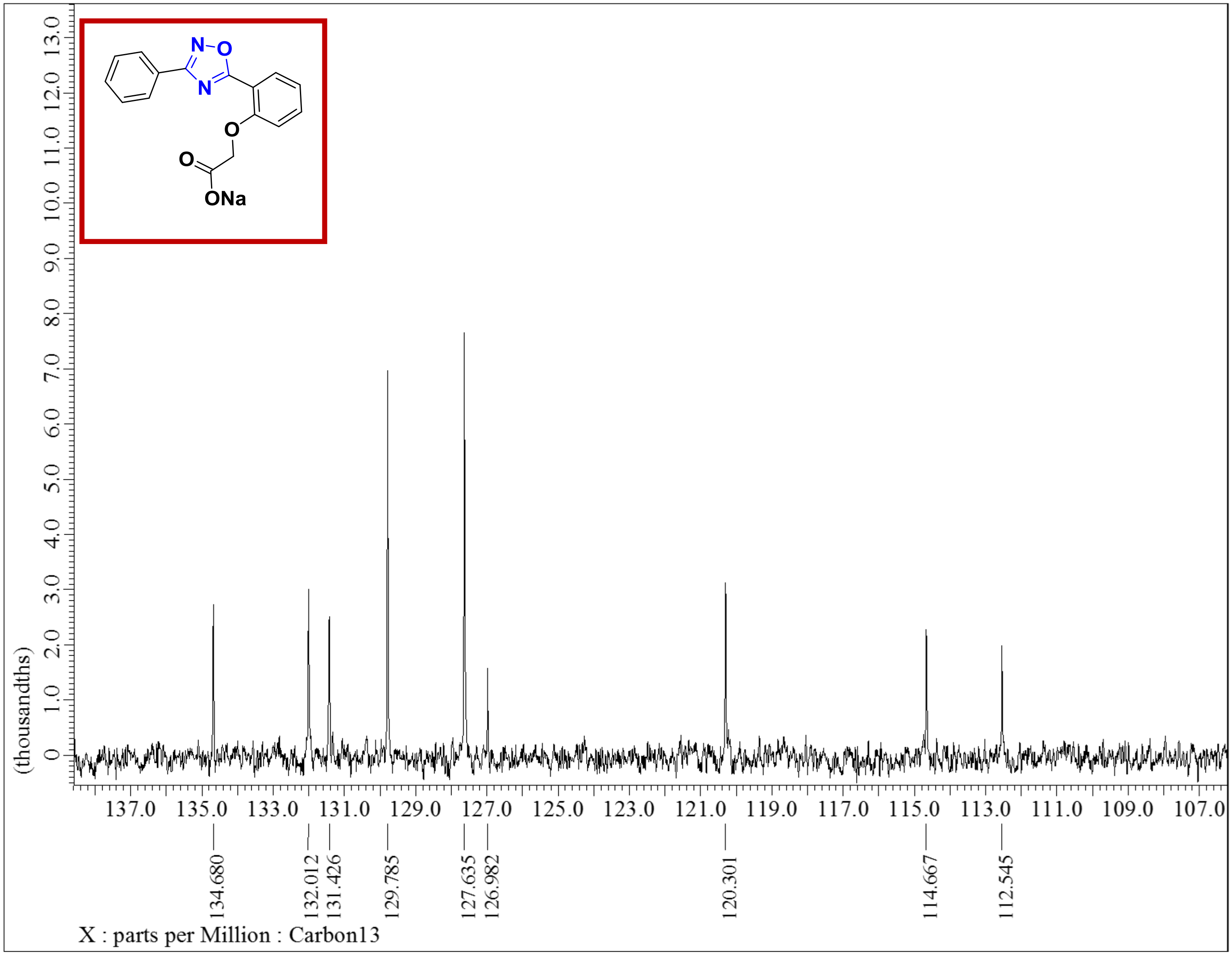

Fig. SI.4 *^{13}C-NMR spectrum (125 MHz, DMSO-d$_6$) of **5**.*

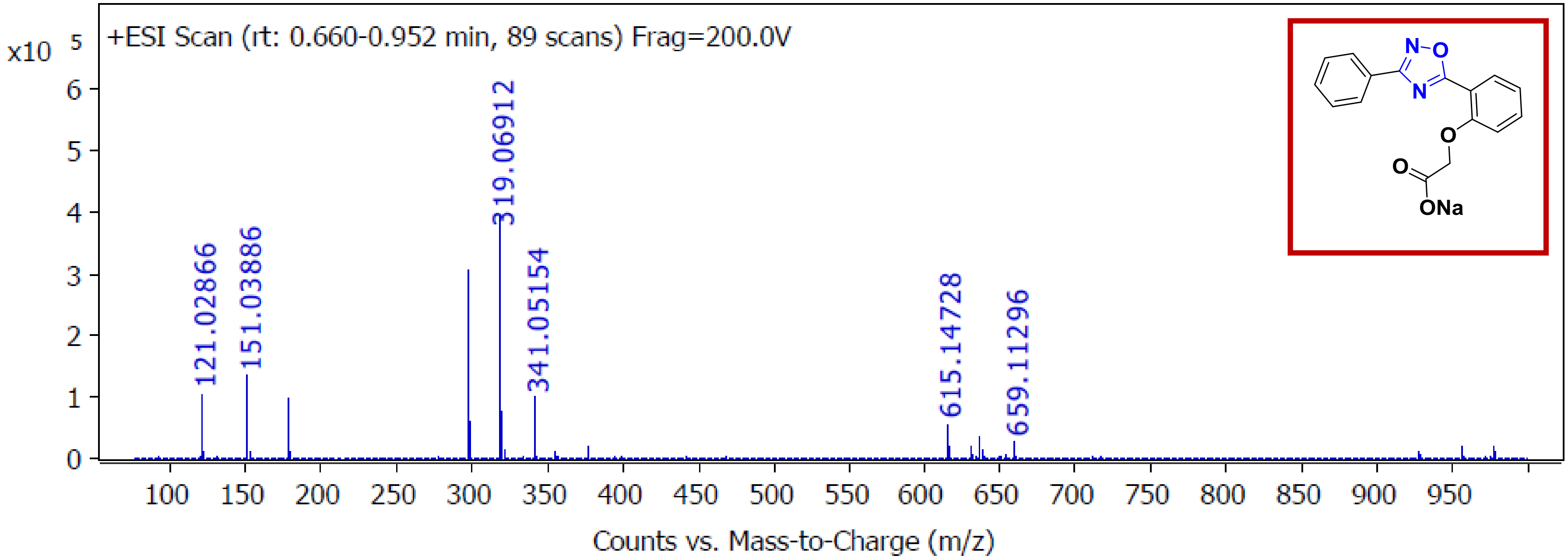

Fig. SI.5 *Mass spectrum of* ***5****.*
